# A High Resolution Genome-Wide Scan for Significant Selective Sweeps: An Application to Pooled Sequence Data in Laying Chickens

**DOI:** 10.1371/journal.pone.0049525

**Published:** 2012-11-29

**Authors:** Saber Qanbari, Tim M. Strom, Georg Haberer, Steffen Weigend, Almas A. Gheyas, Frances Turner, David W. Burt, Rudolf Preisinger, Daniel Gianola, Henner Simianer

**Affiliations:** 1 Animal Breeding and Genetics Group, Department of Animal Sciences, Georg-August University, Göttingen, Germany; 2 Institute of Human Genetics, Helmholtz Zentrum München, Neuherberg, Germany; 3 Institute of Bioinformatics and Systems Biology, Helmholtz Zentrum Müchen, Neuherberg, Germany; 4 Institute of Farm Animal Genetics, Friedrich Loeffler Institut, Neustadt-Mariensee, Germany; 5 The Roslin Institute and Royal (Dick) School of Veterinary Studies, University of Edinburgh, Edinburgh, United Kingdom; 6 Lohmann Tierzucht GmbH, Cuxhaven, Germany; 7 Department of Animal Sciences, University of Wisconsin-Madison, Madison, Wisconsin, United States of America; North Carolina State University, United States of America

## Abstract

In most studies aimed at localizing footprints of past selection, outliers at tails of the empirical distribution of a given test statistic are assumed to reflect locus-specific selective forces. Significance cutoffs are subjectively determined, rather than being related to a clear set of hypotheses. Here, we define an empirical *p*-value for the summary statistic by means of a permutation method that uses the observed SNP structure in the real data. To illustrate the methodology, we applied our approach to a panel of 2.9 million autosomal SNPs identified from re-sequencing a pool of 15 individuals from a brown egg layer line. We scanned the genome for local reductions in heterozygosity, suggestive of selective sweeps. We also employed a modified sliding window approach that accounts for gaps in the sequence and increases scanning resolution by moving the overlapping windows by steps of one SNP only, and suggest to call this a “creeping window” strategy. The approach confirmed selective sweeps in the region of previously described candidate genes, i.e. *TSHR*, *PRL, PRLHR, INSR*, *LEPR, IGF1*, and *NRAMP1* when used as positive controls. The genome scan revealed 82 distinct regions with strong evidence of selection (genome-wide *p*-value<0.001), including genes known to be associated with eggshell structure and immune system such as *CALB1* and *GAL* cluster, respectively. A substantial proportion of signals was found in poor gene content regions including the most extreme signal on chromosome 1. The observation of multiple signals in a highly selected layer line of chicken is consistent with the hypothesis that egg production is a complex trait controlled by many genes.

## Introduction

‘Selection signatures’ are defined as regions of the genome that harbour functionally important sequence variants and therefore are or have been under either natural or artificial selection. The physical extent of such signatures, up- and downstream of the functional variant, is a consequence of the so-called hitchhiking effect. As stated by Maynard Smith and Haigh [1], three patterns are generated locally around the position of a favorable mutation. First, the density of segregating sites decreases in adjacent regions so that the level of variability will be reduced [2], [3]. Second, the site frequency spectrum (SFS), which describes the frequency of allelic variants, shifts from its neutral expectation towards a relative excess of extreme (rare or high) frequencies [4], [5]. Third, a specific linkage disequilibrium (LD) pattern emerges around the target of positive selection relative to what is expected under neutrality [6], [7].

The search for molecular signatures of positive selection has been a matter of intense research in recent years, motivated by the hope to associate genes that experienced recent strong selection with functions and phenotypes (for review see [8], [9]. These studies have resulted in the development of various statistics aimed to detect selection at the DNA level in population samples. The methods used are based either on the site frequency characteristics (focusing on single loci) or on properties of haplotypes segregating in populations.

In site frequency based methods the level of DNA polymorphism is assessed for a very large number of loci on a genome-wide scale within populations. Conceptually, the goal is to identify genomic regions with a reduced variation or a different shape of the SFS than the norm of the genome. These methods essentially assume that demographic effects and population structure affect the whole genome in the same fashion; on the other hand positive selection should influence only individual genes and, through the hitchhiking affect, the surrounding regions. This concept has been used on a genome-wide scale to detect signals of past selection in humans and other species [10]–[15]. Genomic scans for local variability have also been conducted in chicken [16], [17]. The last authors introduced the so-called “Pooled Heterozygosity” (*H_P_*) statistic, a variability estimator based on allele counts across sliding windows of adjacent loci to look for areas that deviate from the norm.

It is important to note that many of these studies have focused on the observed distribution of a given test statistic, assuming that loci in the tails of this distribution have been targets of recent selection [18]. Although this approach to detect selective sweeps in genome-wide data sets seems appealing, questions about the statistical validity of this strategy have been raised [19], [20], [9]. Since, as highlighted by Williamson et al. [21], the prevalence of selection in the genome is unknown, the “empirical *p*-value” strategy does not directly test the hypothesis of selection at any putative locus and provides no means for quantifying how common selection is across the genome. For instance, the null hypothesis of selective neutrality could be true for the entire genome, in which case even the most extreme values would carry no information regarding selection.

Kim and Stephan [22] proposed the composite likelihood ratio (CLR) test to localize selective sweeps in subgenomic regions based on the change in the shape of the allele frequency spectrum. They used coalescent simulations to derive a distribution of the test statistic under the null hypothesis of no selection. However, the use of simulation requires accurately mimicking population demography as well as making assumptions that may or may not hold (e.g., uniform recombination or mutation rate across the genome, etc). In a similar study Nielsen et al. [23] extended the CLR test to derive the expected background pattern of variability from the data itself, rather than from a population genetic model. This approach compares a neutral null model for the evolution of a genomic window with a selective sweep model and can be applied to species having sufficient genome wide SNP data available [7], [21], [24]. It appears that CLR is one of the few metrics that robustly tests the statistical significance of a putative region for the hypothesis of positive selection.

In this study, we compared genome-wide *H_P_* estimates based on 2.9 million SNPs from a commercial line of egg laying chickens (see methods). We employed a modified sliding window approach (referred to as a “creeping window”, CW) and validated the method by confirming the identification of previously described candidate genes. Furthermore, we used a permutation method that uses the original allele frequency spectrum of the genome under study to define the significance thresholds for the *H_P_* values. In total 132 genes or genomic regions that display patterns of genetic variation consistent with the hypothesis of positive selection are presented, comprising some striking examples of selective sweeps that span over several megabases.

## Results and Discussion

### Creeping windows

**Figure 1:**
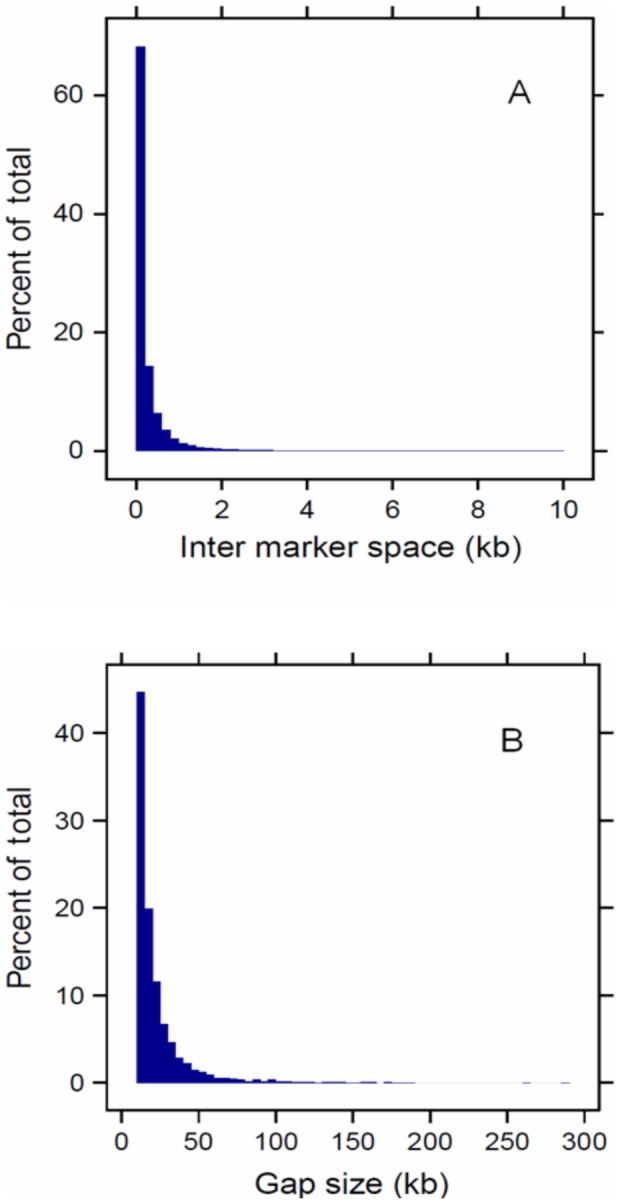
Histograms of (A) distance between neighboring markers and (B) gap size in the final data set.

Scanning a genome by sliding a non-(or partly) overlapping window of uniform length along the sequence is a common strategy in site frequency based methods. The primary objective of such “sliding windows” (SW) strategies is to reduce the noisiness of single-locus statistics by combining data from several adjacent markers. The window size is often subjectively determined which can influence the final results and interpretations. Regarding the fact that continuous stretches of *H_P_* values (or any site frequency based metric) are correlated to an extent determined by the level of linkage disequilibrium, one may suggest adjusting window sizes such that the extent of LD is reflected [25]. However, it remains unclear how to account for the age of selective sweeps in view of their diverse length, as well as for varying levels of local LD across the genome or between populations. The CW method we used (see Methods) has the advantage of simplicity and is applicable with all site frequency based statistics. In addition, the algorithm accounts for the non-uniform distribution of markers, so that artifact signals originating from conflicting effects of genomic gaps are avoided. Figure 1 illustrates lack of uniformity in the distributions of inter-marker distance and gap size.

**Figure 2:**
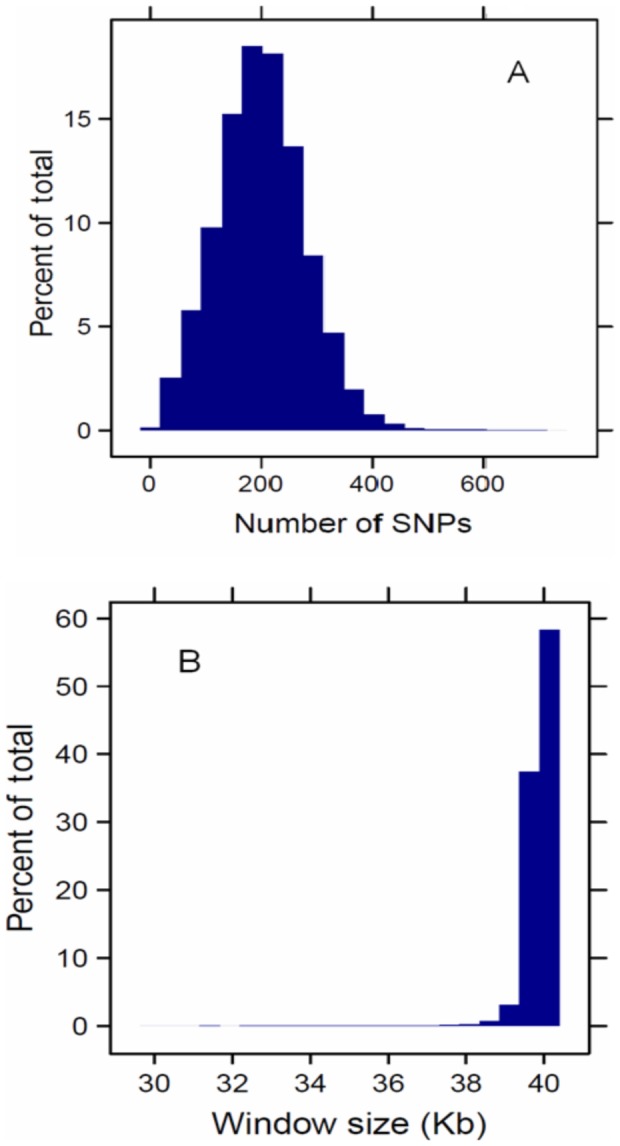
Distributions of (A) the number of SNPs per window and (B) the size of 862’400 windows creeping along chicken chromosomes GGA1 to GGA28.

To evaluate the performance of the CW approach the distribution of pooled heterozygosity profiles was compared with different implementations of the SW approach. Applying the CW strategy with *H_P_* values genome-wide resulted in 862’400 windows. The mean number of SNPs in a window was 199±78 with window size varying between 30 and 40 Kb, having a median of 39’909 bp (Figure 2). While 40 Kb was the specified standard, shorter windows may result from gaps in the CW approach.

**Figure 3:**
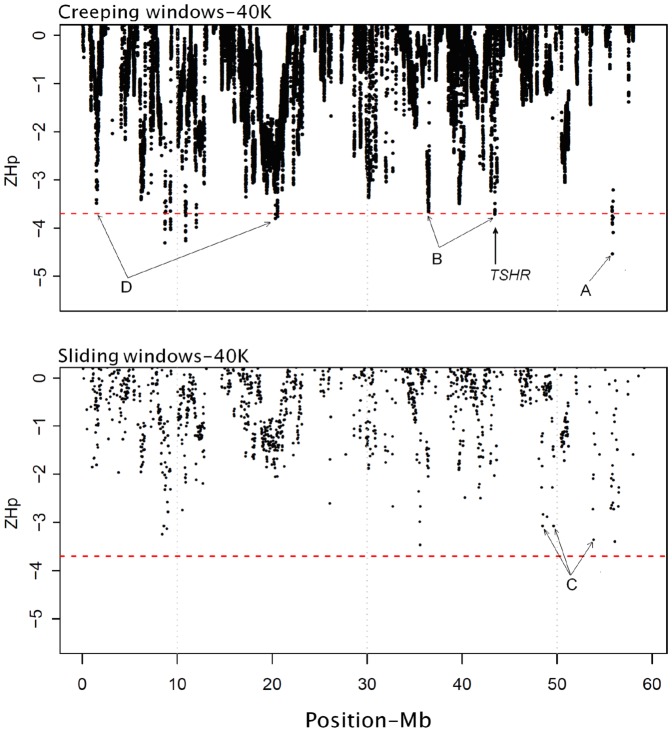
The negative end of the *ZH_P_* distribution from the creeping windows (CW) versus the sliding windows (SW) strategy is presented along GGA5. The horizontal dashed line stands for the significance level at *P*≤0.001 (*ZH_P_* = −3.70, genome-wide) and vertical gridlines help to compare similar signals between plots. Capital letters highlight positions where (A) CW produces more pronounced signals than SW; (B) SW misses signals found by CW; (C) SW produces spurious signals not confirmed by CW; and (D) CW finds classic long-range sweeps with typical patterns. A strong signal is found at the position of the *TSHR* gene already described by Rubin et al. (2010).

In the SW scenarios we walked through the genome with non- or partly overlapping windows of size 40 Kb in steps of 0 to 20 Kb, respectively (data are shown only for the 20 Kb overlapping scenario). A panel of 40’289 windows in the partly overlapping scenario was created, which is explicitly a function of the extent of overlap between consecutive windows. Figure 3 illustratively compares the negative end of the *ZH_P_* distribution for 61’538 creeping versus 2658 sliding windows, respectively, across chromosome GGA5.

The comparison of the two profiles shows the following main discrepancies:

- The magnitudes of extreme signals obtained with the CW approach are higher than those obtained with the SW approach (e.g. at position A);
- The CW approach reports clear signatures of selection that are missed by the SW approach (e.g. at position B)
- The SW approach produces some spurious signals that are not confirmed by the CW analysis as these may be artifacts caused by gaps in the sequence (e.g. at position C)
- The CW approach identifies clear stretches of a selective sweep, with a typical gradient of decreasing *ZH_P_* values from both sides, which is much less pronounced in the SW approach (e.g. at position D)

These examples highlight the possibility that some selection signatures may have been missed or erroneously accounted for in previous studies based on SW approaches. In general, our results indicate an improved efficiency in signal detection for scanning genomes with the CW strategy. However, it must be noticed that intensified resolution sharply enlarges the number of windows, which affects the multiple testing issue.

With the example of chromosome GGA5 (Figure 3) the ability to detect a selection signature using creeping windows of 40 kb is confirmed by localizing the previously described *TSHR* gene [17], along with two typical selective sweeps of different size depicting valleys of heterozygosity (D). One distinct sweep is observed at chromosomal position 19.3 to 21’5 Mbp harboring the *APIP*, *PDHX*, *CD44*, *ACTC1*, *GPIAP1*, *NAT10*, *RAG1* and *RAG2* genes. Another evident sweep is spanned over 1.27 to 1.72 Mbp overlapping the *IGHMBP2*, *SYT12*, *Cor6*, *SIRT3*, *RIC8A*, *NADSYN1* and *ZDHHC13* genes.

### Revealing genome-wide significant signals

Faced with problems in determining the null distribution of a test statistic, researchers often focus on top-ranking SNPs and avoid specifying testable hypotheses. However, an outlier locus is not necessarily indicative of selection. In such an approach there are basically no a priori criteria available for deciding how extreme a region needs to be in order to claim a selection signal and the significance cutoffs are determined subjectively, rather than being derived from a model. Using permutation re-sampling in this study we derived a null distribution for testing the genome-wide significance under the null hypothesis of absence of selection (see Methods). Briefly, this permutation method maintains the original structure observed in the real data set such as the SNP density, and the background distribution of *H_P_* values is computed after the frequencies of the SNPs are shuffled.

**Figure 4:**
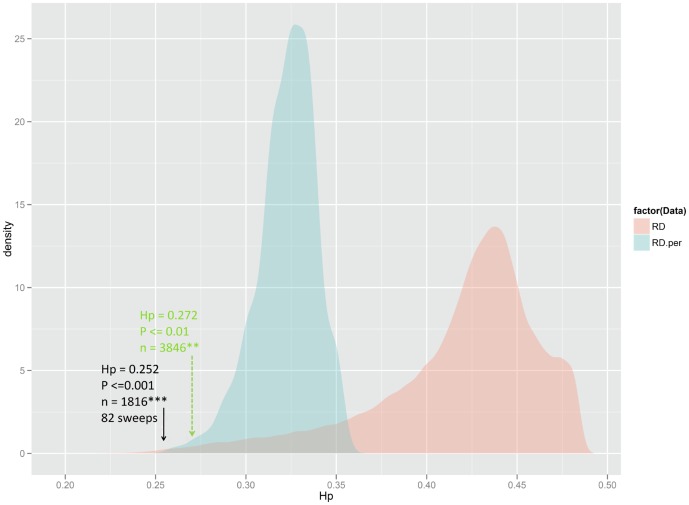
Distribution patterns of the *H_P_* profile from 862’400 windows creeping over the genome. Pink and blue densities represent, respectively, the observed and the panel of recorded lowest *H_P_*-values from 10’000 re-sampling runs in real data. Windows with *H_P_*≤0.252 represent significant signals at the empirical error level *P*≤0.001. As indicated, 1816 windows characterize 82 selected regions with a more extreme local homozygosity than expected under neutrality.

Evidence of positive selection was investigated by assessing variation in allele frequency across the genome. In total, 862’400 windows were tested. The mean *H_P_* value was estimated as 0.418±0.045 and the lowest *H_P_* was 0.196 for a region on chromosome GGA1. Figure 4 compares the distribution of *H_P_* values from the observed data against the profile of the lowest *H_P_* values recorded in each permutation. As shown, the lower limit of *H_P_* values obtained from 10’000 permuted datasets was 0.250 whereas the lowest *H_P_* value from real data was 0.196. Accordingly, we placed the critical value for claiming candidate selective sweeps with an empirical genome-wide significance level *P*≤0.001 at *H_P_* = 0. 252 (*ZH_P_* = −3.70) and windows below this threshold were considered to represent selection signals. In total, 1816 putative windows, many of them overlapping, with a statistically significant (*P*≤0.001) departure from the norm of allelic variability were observed. This number exceeds the number obtained when we just accept the 0.1 per cent smallest (i.e. 862) values, as done in the usual outlier approach. However, with less stringent thresholds on the empirical *p*-values, for instance *p*≤0.01 (*ZH_P_*<−3.50, genome-wide), only 3846 significant windows are obtained, which is considerably less than the 1% (8624) top-ranked signals.

A striking feature that emerges when examining the distribution of allelic variability via the CW strategy is that diversity values tend to cluster together. This results in consistent signatures of selective sweeps for adaptive alleles, which in some cases extend over stretches of several megabasepairs. We considered these adjacent signatures as “distinct” if they typically exhibited the pattern of decaying *H_P_* by distance to both sides (cf. position D in Figure 3).

Across the genome, we counted signals of pooled heterozygosity (*P*≤0.001) that were accompanied by at least two consecutive significant windows (*P*≤0.05, genome-wide). In total, we observed 82 clusters representing strong evidence of selective sweeps. However, we believe that additional loci further down the list deserve closer examination in follow-up studies. The number of detected regions rose to 132 when the significance threshold of pooled heterozygosity was set to *P*≤0.01. Table S1 presents test statistics including the number of signals on each chromosome and positions for the full panel of regions that fell below *H_P_* = 0.272 (*P*<0.01, genome-wide). The observation of multiple signals in a commercial layer line is consistent with the hypothesis that egg production is a complex trait controlled by many genes.

In order to visualize the chromosomal distribution of significant signals, we plotted the *ZH_P_* statistic against genomic position (Figure 5). Furthermore, a detailed graphical representation of the *ZH_P_* signals for the 28 autosomes is reported in supporting information (Figures S1, S2, S3, S4, S5, S6, S7, S8, S9, S10, S11, S12, S13, S14, S15, S16, S17, S18, S19, S20, S21, S22, S23, S24, S25, S26, S27, S28). It is evident that the signals are non-uniformly distributed across chromosomes and chromosome segments.

**Figure 5:**
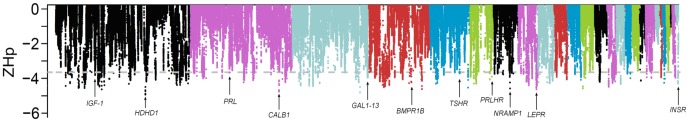
The negative tail of the *ZH_P_* distribution presented along GGA1 to GGA28. Each dot represents a CW of 40 Kb and arrows point at the location of candidate genes (Table 1) and genes with reported associations in the literature. The horizontal dashed line indicates the significance threshold at *P*≤0.001 (Genome-wide *ZH_P_* = −3.7).

### Simulation

To evaluate the performance of our method for delimiting the significance of a selective sweep, we performed computer simulations. We considered models involving both neutrality (Neut) and a selective sweep (SP) at a single locus. The genomic distribution of SNPs and selective sweeps (i.e., one Sweep per 10 Mb) in the simulation scheme corresponds roughly to the chicken genome analyzed with the current SNP array. Two *H_P_* sets with 23’265 and 22’955 windows were calculated in the Neut and the SP models, respectively. The mean Neut*_HP_* was estimated as 0.282±0.014 with a minimum of 0.234 which dropped to 0.224 in the sweep scenario. Figure 5. a, b respectively, depicts the profile of *H_P_* values in the Neut and the SP simulations along with the location of the selective sweep in the middle of the simulated chromosome.

**Figure 6:**
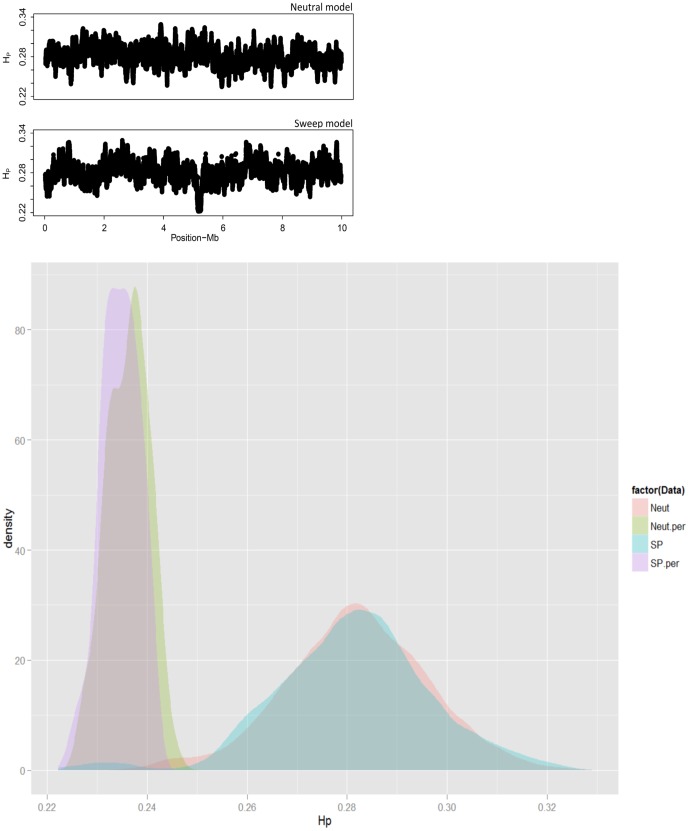
A graphical representation of simulation results from two genetic models. Neut and SP abbreviate genetic models with neutral and selective sweep along with Neut.per and SP.per representing corresponding distribution of permutation. *H_P_* profiles estimated from 23’265 and 22’955 creeping windows are plotted across a chromosome of 10 Mb in Neutral (a) and Sweep (b) models, respectively. A distinct valley of homozygosity at the middle of chromosome represents the simulated sweep. (c) Density distributions of *H_P_* profiles form both models are depicted along with 1000 resamples.

We applied our test statistic to both simulated data sets. For this, allele frequencies from each data set were randomly shuffled across chromosomal positions and a profile of the smallest *H_P_* values was generated from 1000 iterations. To compare the distribution of *H_P_* profiles, we plotted the kernel density from both scenarios against their minimum profiles from permutations (Figure 6. c). As shown a perfect overlap is evident between both simulations and corresponding permutations except for *H_P_* windows representing the selective sweep. The smallest *H_P_* value from the neutral simulation is 0.234 which is distant from the minimum *H_P_* value from permutations (0.226). Therefore, the test correctly assigns a non-significant *p*-value≤0.62 to the lowest heterozygosity window of the neutral simulation. On the other hand, in the selection scenario, the lower limit of SP*_HP_* = 0.224 was only exceeded in three permutation resamples with a minimum of *H_P_* = 0.222. This signifies that the simulated sweep is conservatively detected at a significance level of *p*≤0.003 (SP*_HP_* = 0.224).

### Validation with candidate genes

The thyroid-stimulating hormone receptor (*TSHR*) gene, a well-documented example of positive selection in the chicken [17] was used as a positive control to examine the validity of our approach. We extended the analysis to additional candidate genes known to be related with production traits and, therefore, being potentially under positive selection. For instance, the insulin-like growth factor 1 (*IGF1*) is known to be associated with growth, body composition, and skeleton integrity in chickens [26], [27]. Candidate genes were identified from the literature and databases including *NRAMP1*, *PRL, PRLHR, INSR*, *LEPR* and *IGF1*. We could not include *GHR, PRLR* and the *BCDO2* gene causing yellow skin colour in our validation panel because they were either located on chromosome Z or the SNP coverage in the corresponding regions was not sufficient to effectively test the variability of these regions. The regions surrounding the genes displayed an elevated homozygosity compared to the genome-wide average. Table 1 presents the names, position and summary statistics for the chosen panel.

**Table 1:** Summary statistics of the pooled heterozygosity metric for selection signature in candidate genes.

Gene	Chr	Position-bp	*H_P_*	*P≤*	Function/association	
*IGF1*	1	57’327’750..57’376’178	0.24	0.001	Key regulator of muscle development and energy metabolism in birds.	[17], [26], [27], [28]
*PRL*	2	59’724’582..59’730’725	0.26	0.01	Egg laying pattern and production	[28]
*TSHR*	5	43’202’356..43’250’961	0.25	0.001	Inhibitory effect on Growth hormone secretion	[17]
*PRLHR*	6	31’242’680..31’243’785	0.24	0.001	Governing early embryonic axis formation	[29]
*NRAMP1*	7	24’283’380..24’363’380	0.21	0.001	Natural resistance to Salmonella infection and macrophage function	[30], [31]
*LEPR*	8	29’125’599..29’156’553	0.21	0.001	Affecting feed efficiency	[32]
*INSR*	28	3’431’232..3’471’081	0.27	0.01	Insulin signaling	[17]

The results revealed a significantly different *H_P_* profile in most of the candidate regions. For example, a window perfectly overlapping the *IGF1* gene on GGA1 represents the most extreme signal in the corresponding region. Consistent with Rubin et al. [17], we further observed locally reduced variation for a short region surrounding the *TSHR* gene on GGA5 (Figure 3). In contrast, some regions contained several consecutive windows with consistently low *H_P_* values. For instance, the Leptin receptor gene (*LEPR*), a candidate gene with a central role for Leptin signaling affecting feed efficiency, displays statistically significant low-*H_P_* windows extended over several Mb (Figure S8), possibly indicating that this locus has been subject to recent selective pressures. In addition to the age of selection, several factors may affect the size of a selective sweep, like the local recombination rate, whether the selected variant ever reached complete fixation, the number of generations it took before fixation and any population admixture at a time point after the sweep initially occurred.

### Functional annotation of regions under selection

We annotated the genomic regions harboring significant signals using the map viewer program, and by aligning the positions to the second draft of the chicken genome sequence assembly, to reveal genes and ESTs located in the respective region. Table 2 summarizes statistics for a collection of selected regions across the genome harboring the strongest signals along with the distinct sweeps. The window with the smallest *H_P_* value (*H_P_* = 0.196, P<0.001) was observed on GGA1 embedded within 130’539’515 to 130’579’189 bp. This is a poor gene content region with no coding sequence mapped. The region, however, depicts the pattern of a distinct sweep spanning over 2 Mb (Figure S1). We extended the window to its decaying domains in both directions up to 700 kb to find the biologically most interesting candidate gene in this region. Of the 8 ESTs in this region, haloacid dehalogenase-like hydrolase domain containing 1A, was the only gene in the region. *HDHD1* is a conserved gene in many species and very little is known about its biological importance. Another strong signature of selection on GGA2 (*H_P_* = 0.203, P<0.001) matched the Calbindin 1 gene. *CALB1* is a 28,000-kDa calcium-binding protein, which fluctuates in a circadian fashion during the daily egg cycle, in close temporal association with eggshell calcification [33], [34]. It was shown that the pattern of *CALB1* expression is related to eggshell quality [33] and eggshell abnormalities in layer chickens [35]. Association was also demonstrated between *CALB1* gene expression and reduction of eggshell thickness after xenoestrogen treatment [36]. Moreover, on chromosome 4, a region harboring the secreted phosphoprotein 1 or Osteopontin gene showed a signal of positive selection (*P*-value<0.01). It was suggested that *SPP1* could be involved in the mechanism controlling the arrest of eggshell calcification [37] and the specific occlusion of *SPP1* into calcite during mineralization may influence eggshell structure and thereby modify its fracture resistance [38]. There are also reports that polymorphisms within the Osteopontin gene are associated with 5-week body weight in egg laying chickens [39]. Further to the strong signal overlaying the *Nramp1/SLC11A1* gene, which is a well documented candidate for immune traits in chickens, a distinct sweep (P<0.001) was detected on chromosome 3 embedding the gene cluster Gallinacin 1–13. This cluster is designated densely within a 86-Kb distance and encodes Avian beta-defensins, a family of antimicrobial peptides that are capable of killing a broad spectrum of pathogens and play a critical role in innate immunity in chickens [40]. Beta-defensins are also present in different compartments (eggshell, egg white, and vitelline membranes) of the egg and are expected to be involved in the protection of the embryo during its development and to contribute to the production of pathogen-free eggs [41].

**Table 2:** Collected panel of genomic regions identified as candidate selective sweeps.

Chr	Position^a^	*H_P_*	*P*	Function^b^	Gene
1	*129’979’844..* *132’096’418*	0.19	0.001		*HDHD1A*
2	77’468’964..81’953’567	0.22	0.001		*FASTKD3, CCT5, CMBL, ROPN1L, DAP, ANKH*
2	*127’197’645..* *129’637’001*	0.20	0.001	Eggshell abnormalities	*CALB1*
3	98’653’321..98’693’223	0.20	0.001		
3	109’525’540..110’278’233	0.24	0.001	Production of pathogen-free eggs	*GAL1-13*
4	9’972’965..12’799’763	0.24	0.001		*SOX3, GABRB1*
4	*47’835’606..* *48282844*	0.25	0.01	Eggshell fracture resistance, body weight	*SPP1*
4	50’359’254..51’663’078	0.23	0.001	Regulates the activity of IGF1, 2 genes	*IGFBP*
4	59’893’444..60’932’043	0.22	0.001	Major determinant of Litter size in sheep	*BMPR1B*
6	64’439..639’455	0.24	0.001		
5	1’273’963..1’717’264	0.26	0.01		*IGHMBP2, SYT12, cor6, SIRT3, RIC8A, NADSYN1, DHHC13*
5	19’289’172..21’554’715	0.24	0.001		*APIP, CD44, ACTC1, GPIAP1, NAT10, RAG1, RAG2*
10	3’595’855..4’183’588	0.24	0.001		*HMG20A, LRRN6A, RCN2*
12	5’756’755..6’744’249	0.24	0.001		*WNT7A, BARX1, MIRNLET7D, MIRNLET7F, MIRNLET7A-1, HDAC11*
12	16’889’808..17’356’279	0.26	0.01		*SHQ1, PPP4R2, PDZRN3*
20	1’630’787..2’136’985	0.24	0.001		*EIF2S2, CHMP4B, E2F1, CBFA2T2*
20	5’659’382..7’543’732	0.26	0.01		*CSE1L, STAU1, CCNDBP1, PPP1R3D, EYA2, SULF2, CDH4*

a Positions in normal format represent “distinct sweeps” revealed by the *H_P_* metric. A distinct sweep spans over numerous consecutive significant windows and depicts a typical valley of heterozygosity.

b Signals overlapping genes with a previously described association.

Some of the regions identified contain genes with biological functions that were previously discussed in connection to traits under selection in other species. For example, strong evidence of a sweep reflected by a set of windows on GGA4 (P<0.001) involves the bone morphogenetic protein receptor, type IB gene (*BMPR1B*) which is a major determinant of ovulation rate and litter size in sheep [42], [43]. A candidate gene affecting growth traits and with a central role in regulating IGF gene, insulin-like growth factor binding protein (*IGFBP*), also lies within a distinct sweep region on GGA4 (Table 2). We also found several other regions harboring genes with biological functions that could be related to (production) traits. In general the annotation list (Table S1) is enriched with genes of biological interest involved in carbohydrate metabolism pathways, muscle-skeletal structure development, solute carrier proteins, calcium signaling pathways and the immune system.

The first genome-wide scan of selection for local homozygosity in the chicken was performed by ICPMC [16] using sequence data from only 3 individuals representing layers, broilers and the Red Jungle fowl, respectively. In a more comprehensive study, Rubin et al. [17] re-sequenced pooled DNA from a number of commercial and domestic lines to identify selective sweeps of favorable alleles. Local heterozygosity was calculated in sliding windows of 40 Kb, and seven putative selective sweeps were detected in layers at 6 standard deviations away from the genome mean. In addition to the aforementioned candidate genes *TSHR*, *INSR* and *IGF1*, two out of seven regions overlapped with regions revealed in the present study. Identification of these regions in two independent studies supports the hypothesis that these regions have strong signatures of selection and are likely to be true positives.

There are, however, several regions with strong evidence of selection identified in our study that were not reported previously. Apart from genetic drift, the differences may result in part either from the insufficient power of the tests employed or from insufficient coverage in the datasets scanned. The SNP calling depth in the current study was at least four times larger than the one in earlier studies, which provides more reliable allele frequency estimates. As demonstrated above, the scanning resolution in the CW approach is much better than the one obtained with SW, which raises the possibility that some signals may have been missed or falsely reported in previous studies (cf. Fig. 3). The inconsistencies can also originate from the lack of a consensus threshold in empirical approaches. Earlier studies just reported a fraction or the most extreme results (i.e. the 1% or 0.1% outliers in the empirical distribution), while in our study a permutation-based genome-wide significance threshold was applied. Combining this conservative testing strategy with the identification of candidate regions (characterized by a series of significant windows) yielded a relatively low number (132) of significant regions for selective sweeps (listed in Table S1) albeit of high credibility. Finally, there are signals that probably do not reflect historic selection at all, but rather arise from local genomic differences in mutation or recombination rates, or are statistical outliers in multiple genome-wide tests for significance.

## Conclusions

We adapted a permutation-based re-sampling method as a valid approach to test the significance of differences in local variability. The method uses the original allele frequency spectrum of the genome under study to maintain the observed SNP structure for defining an empirical *p*-value. However, it assumes a uniform demography across the genome and generates the null distribution based on independence of allele frequency estimates between neighboring SNPs which is violated in a real scenario. We realize the permutation approach to testing for significance is very straightforward, and it may be argued that more sophisticated methods could generate a null distribution by performing neutral simulations with a range of demographic and recombination effects. However this bears its own challenges in defining the models appropriately such that they reproduce the full SNP structure in the data set, and even then we are not certain it would yield greater sensitivity or specificity in detecting sweeps. We also improved the resolution of signal detection using a creeping window strategy. Genome-wide, 82 regions with strong evidence of selection (*P*-value<0.001) were identified including genes known to be associated with eggshell quality and immune system, such as *CALB1* and the *GAL* cluster. Our results confirm the presence of selective sweeps in regions of previously described candidate genes, in some cases spanning over intervals of several megabases. The observation of multiple signals is consistent with the hypothesis that egg production is a complex trait controlled by many genes. The major challenge remains to distinguish true signals from those due to genetic drift. One possible solution involves analyzing separate populations with different phylogenetic history, but selected for similar breeding goals (e.g. white-egg layers and brown-egg layers), hypothesizing that true signals generated by selection would overlap across the populations. Such efforts are currently underway by the authors, along with validation of results obtained with other methods of selection signature detection. Further research should also try to verify hypothesized relationships between gene networks rather than single genes underlying the observed pattern of selection signatures. Our results may be of future interest for identifying signatures of artificial selection in commercial chicken breeds or as additional evidence for any polymorphism that shows associations with egg production traits.

## Materials and Methods

### Ethics statement

Samples were collected by veterinarians in the Lohmann company in the course of a routine health check for diagnostic reasons and a partition of these samples was used to extract DNA. The authors collected no samples themselves.

### Whole genome re-sequencing and SNP discovery

We studied a commercial brown layer line provided by Lohmann Tierzucht GmbH. Blood samples were collected with EDTA as anticoagulant from the wing vein of 15 unrelated female birds originating from different sire families. DNA was extracted from blood samples following a standard Phenol/Chloroform extraction protocol [44]. DNA quality and concentration of each sample was calculated and equal amounts of DNA of 15 samples were mixed to produce the DNA pool for sequencing.

Sequencing libraries were constructed with paired-end DNA sample preparation kits (Illumina) according to the manufacture's recommendations. Sequencing was carried out on an Illumina Genome Analyzer IIx as 76 bp paired-end reads. We sequenced two lanes of a flow cell yielding 22.0 Gb reads. Image analysis and base calling was performed using the Genome Analyzer Pipeline software.

Sequence reads were mapped against the third build of chicken reference genome (yet to appear officially in the public databases). Prior to mapping, the reference genome was repeat masked using RepeatMasker. To further remove potentially problematic areas of the genome, 16 mers occurring more than 5 times were also masked. Reads were aligned to the reference genome using BWA version 0.5.7 with default parameters. Samtools version 0.1.7 [45] was used to remove potential PCR duplicates and to call SNPs. About 112.0 million reads aligned to the genome with a mapping quality score of 20 or more. A SNP was called when the position was covered by at least 5 reads with a mapping quality score of 20 or over, and a base quality score of 20 or over.

### Quality control and data filtering

The total number of SNPs detected in the pool was 4’540’269. We checked the markers for redundant positions and applied a number of rules to edit SNPs for further analysis. To minimize incorporating false SNP, we used Phred scaled SNP quality score, Q, which is related to the SNP calling error probability (*p*) by the equation: Q = −10×log_10_(*p*). The average SNP quality score was estimated as 100±50. We kept polymorphisms with a minimum score of 20 (99% accuracy) as an acceptable error rate (Figure S29 in the Online Data Supplement).

Polymorphisms detected had a read depth between 1 and 20’895. To reduce potential errors in SNP frequency estimates in the pool of 15 individuals, and to preclude over-representation of repetitive sequences, we only used polymorphisms with a read-depth between 15× and 50×. In the final data set the average read depth was 21.9±5.0. Figure S30 displays the distribution of the depth of SNP calling in the final data set analyzed.

Analysis of the inter-marker distance between polymorphisms revealed numerous genomic gaps (regions free of SNPs) on some microchromosomes and chromosome Z of up to 5 Mb or larger. Therefore, only autosomes GGA1-GGA28 were included in the final analysis. In the filtered data the average inter-marker space was estimated as 314.2±1362.1 bp (median = 97 bp), and 5503 gaps were present across the genome. Figure S31 presents a genome wide image of marker distribution in the original SNP panel. The accumulated proportion of genomic gaps was estimated as 13.3% of the genome after filtering.

In total 2’913’540 SNPs on 28 autosomes were included in the final analysis. Average minor allele frequency (MAF) was 0.31±0.11, and only 16’135 markers (0.5%) had a minor allele frequency of less than 10% (Fig. S32). The pattern of MAF distribution was fairly similar to those from already available commercial 37 K and 60 K Illumina bead chips.

### Detecting selective sweeps

To identify genomic regions that may have been targets of past selection, we used the pooled heterozygosity (*H_P_*) statistic suggested by Rubin et al. [17]. For a window with 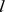 loci,
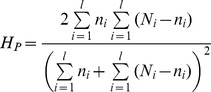
where 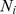 is the number of reads at locus 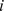 and 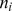 is the number of reads of the most abundant allele at locus 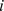. *H_P_* values were z-transformed to *ZH_P_* values with mean = 0 and SD = 1 to facilitate visualization of the outlying signals and comparison with previous reports.

### Sliding and creeping window approach

**Figure 7:**
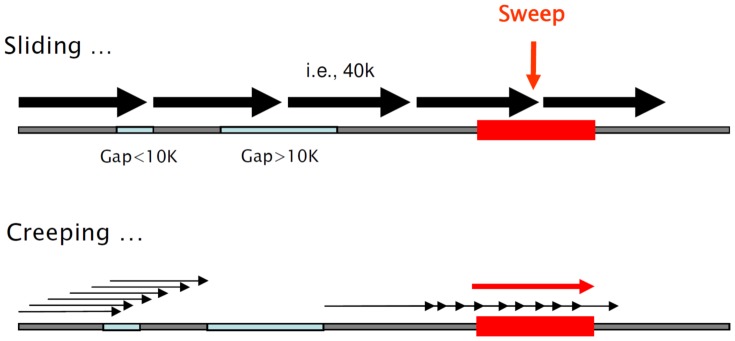
A graphical comparison of two genome scanning strategies. sliding windows (SW) vs., creeping windows (CW). With SW a chromosome is split into non (or partly) overlapping windows of 40 K and while passing over genomic gaps, it may not perfectly overlie a selective sweep. CW implements an elevated resolution moving windows in steps of only one SNP forward. The approach bridges small (<10 K) gaps while it stops at larger gaps and re-starts at the opposite side. CW always centers a window relative to a sweep position.

To facilitate comparisons of genomic regions with a higher resolution we adopted a more expedient approach called “creeping window” to scan the entire genome for evidence of selective sweeps (Figure 7). This is an intensified “sliding window” strategy that moves windows in steps of only one SNP forward and, while passing over genomic gaps <10 Kb, it skips gaps >10 K and re-starts from the first SNP after a gap. We acknowledge that specifying 40 kb as window size was subjective, but it was motivated from previous studies and by the desire of having a sufficiently large number of SNPs in a window. According to Rubin et al. [17] spurious fixation signals are more likely to occur when few chromosomes are sampled from a DNA pool and inadequate numbers of polymorphic loci in windows are analyzed. Thus to avoid noise in estimates of non-uniform windows we removed windows <30 K and those with less than 10 SNPs for further analyses.

### Assessing statistical significance

We followed the ideas of Churchill and Doerge [46] in applying a permutation approach to define empirical significance thresholds for any individual window. For this, the SNP positions are taken as fixed and allele frequencies are randomly shuffled across positions in each iteration. This is followed by computing pooled heterozygosities for creeping windows of 40 K from the shuffled data and the genome wide lowest *H_P_* from a window ≥30 K formed by ≥10 SNPs is stored. After repeating this procedure for *n* iterations, the empirical threshold pertaining to error probability *P* = 0.001 is the value cutting of the 0.001 quantile in the ordered vector of minima. We ran the simulation with n = 10’000 iterations, computing *H_P_* values for 862’400 creeping windows in each iteration. This approach conserves the genome structure, like SNP densities and gap positions, and allows simulated data to be randomly drawn from the allele frequency distribution of the population under study. Hence, we do not assume any particular population genetic model to generate the background allele frequency spectrum, but the expected background pattern of variability is given by the data.

### Simulation

Program MSMS [47] was used to simulate genomic samples under a coalescent model with mutation, recombination, and constant population size. In the simulations, we assumed two different scenarios: one is the reference population under neutral conditions, and the other is the test population with a single site under positive selection without recurrent mutations. In each model 100 replications of a chromosome of length = 10 Mbp and sample size = 30 was simulated with a selective sweep evolving at the middle of chromosome for the selection model. The list of simulation parameters used is presented in Table 3. We later simulated pooled NGS data from the genomic samples obtained from MSMS by random sampling of 20 chromosomes in each site independently which is an explicit approximation to the average calling depth in the real data set. Allele frequencies from these data sets were then used to estimate the profile of heterozygosity over creeping windows in each single sample and averaged over the number of replications.

**Table 3:** Parameters for the MSMS simulations.

Parameter		Value
Sequence length	*l*	10’000’000 bp
Sample size	*n*	30
Population scaled mutation rate (per site)	*θ*	10^−8^
Population scaled recombination rate (per site)	*ρ*	10^−8^
Effective population size	*N_e_*	10,000
Number of SNPs		50’000 bp

## Supporting Information

Figure S1 **Chromosome wide distribution of variability measured in overlapping windows of 40 k.**
(PDF)

Figure S2 **Chromosome wide distribution of variability measured in overlapping windows of 40 k.**
(PDF)

Figure S3 **Chromosome wide distribution of variability measured in overlapping windows of 40 k.**
(PDF)

Figure S4 **Chromosome wide distribution of variability measured in overlapping windows of 40 k.**
(PDF)

Figure S5 **Chromosome wide distribution of variability measured in overlapping windows of 40 k.**
(PDF)

Figure S6 **Chromosome wide distribution of variability measured in overlapping windows of 40 k.**
(PDF)

Figure S7 **Chromosome wide distribution of variability measured in overlapping windows of 40 k.**
(PDF)

Figure S8 **Chromosome wide distribution of variability measured in overlapping windows of 40 k.**
(PDF)

Figure S9 **Chromosome wide distribution of variability measured in overlapping windows of 40 k.**
(PDF)

Figure S10 **Chromosome wide distribution of variability measured in overlapping windows of 40 k.**
(PDF)

Figure S11 **Chromosome wide distribution of variability measured in overlapping windows of 40 k.**
(PDF)

Figure S12 **Chromosome wide distribution of variability measured in overlapping windows of 40 k.**
(PDF)

Figure S13 **Chromosome wide distribution of variability measured in overlapping windows of 40 k.**
(PDF)

Figure S14 **Chromosome wide distribution of variability measured in overlapping windows of 40 k.**
(PDF)

Figure S15 **Chromosome wide distribution of variability measured in overlapping windows of 40 k.**
(PDF)

Figure S16 **Chromosome wide distribution of variability measured in overlapping windows of 40 k.**
(PDF)

Figure S17 **Chromosome wide distribution of variability measured in overlapping windows of 40 k.**
(PDF)

Figure S18 **Chromosome wide distribution of variability measured in overlapping windows of 40 k.**
(PDF)

Figure S19 **Chromosome wide distribution of variability measured in overlapping windows of 40 k.**
(PDF)

Figure S20 **Chromosome wide distribution of variability measured in overlapping windows of 40 k.**
(PDF)

Figure S21 **Chromosome wide distribution of variability measured in overlapping windows of 40 k.**
(PDF)

Figure S22 **Chromosome wide distribution of variability measured in overlapping windows of 40 k.**
(PDF)

Figure S23 **Chromosome wide distribution of variability measured in overlapping windows of 40 k.**
(PDF)

Figure S24 **Chromosome wide distribution of variability measured in overlapping windows of 40 k.**
(PDF)

Figure S25 **Chromosome wide distribution of variability measured in overlapping windows of 40 k.**
(PDF)

Figure S26 **Chromosome wide distribution of variability measured in overlapping windows of 40 k.**
(PDF)

Figure S27 **Chromosome wide distribution of variability measured in overlapping windows of 40 k.**
(PDF)

Figure S28 **Chromosome wide distribution of variability measured in overlapping windows of 40 k.**
(PDF)

Figure S29 **Phred quality score distribution.**
(PDF)

Figure S30 **Distribution of the calling read of SNPs in final data set.**
(PDF)

Figure S31 **A genome wide inter marker distance between neighboring markers before data cleaning.**
(PDF)

Figure S32 **Frequency distribution of minor allele frequencies involved in final analysis.**
(PDF)

Table S1 **The list of genomic regions likely to be under selection (** ***P*** **<0.01, genome-wide).**
(DOC)

## References

[1] Maynard Smith J, Haigh J (1974) The hitch-hiking effect of a favourable gene. Genet Res 23: 23–35. 4407212

[2] Kaplan NL, Hudson RR, Langley CH (1989) The ‘hitchhiking effect’ revisited. Genetics 123: 887–899. 2612899 10.1093/genetics/123.4.887 PMC1203897

[3] Stephan W, Wiehe THE, Lenz MW (1992) The effect of strongly selected substitutions on neutral polymorphism: Analytical results based on diffusion theory. Theor Pop Biol 41: 237–254.

[4] Braverman JM, Hudson RR, Kaplan NL, Langley CH, Stephan W (1995) The hitchhiking effect on the site frequency spectrum of DNA polymorphisms. Genetics 140: 783–796. 7498754 10.1093/genetics/140.2.783 PMC1206652

[5] Fay JC, Wu C (2000) Hitchhiking under positive Darwinian selection. Genetics 155: 1405–1413. 10880498 10.1093/genetics/155.3.1405 PMC1461156

[6] Kim Y, Nielsen R (2004) Linkage disequilibrium as a signature of selective sweeps. Genetics 167: 1513–1524. 15280259 10.1534/genetics.103.025387 PMC1470945

[7] Stephan W, Song YS, Langley CH (2006) The hitchhiking effect on linkage disequilibrium between linked neutral loci. Genetics 172: 2647–2663. 16452153 10.1534/genetics.105.050179 PMC1456384

[8] Nielsen R (2005) Molecular signatures of natural selection. Annu Rev Genet 39: 197–218. 16285858 10.1146/annurev.genet.39.073003.112420

[9] Akey JM (2009) Constructing genomic maps of positive selection in humans: Where do we go from here? Genome Res 19: 711–722. 19411596 10.1101/gr.086652.108 PMC3647533

[10] Akey JM, Eberle MA, Rieder MJ, Carlson CS, Shriver MD, et al (2004) Population history and natural selection shape patterns of genetic variation in 132 genes. PLoS Biol 2 (10) e286. 15361935 10.1371/journal.pbio.0020286 PMC515367

[11] Glinka S, Ometto L, Mousset S, Stephan W, De Lorenzo D (2003) Demography and natural selection have shaped genetic variation in Drosophila melanogaster: a multi-locus approach. Genetics 165: 1269–1278. 14668381 10.1093/genetics/165.3.1269 PMC1462856

[12] Bamshad M, Wooding SP (2003) Signatures of natural selection in the human genome. Nat Rev Genet 4: 99–111. 12560807 10.1038/nrg999

[13] Sabeti PC, Varilly P, Fry B, Lohmueller J, Hostetter E, et al (2007) Genome-wide detection and characterization of positive selection in human populations. Nature 449: 913–918. 17943131 10.1038/nature06250 PMC2687721

[14] Akey JM, Ruhe AL, Akey DT, Wong AK, Connelly CF, et al (2010) Tracking footprints of artificial selection in the dog genome. Proc Natl Acad Sci 107 (3) 1160–1165. 20080661 10.1073/pnas.0909918107 PMC2824266

[15] Qanbari S, Gianola D, Hayes B, Schenkel F, Miller S, et al (2011) Application of site and haplotype-frequency based approaches for detecting selection signatures in cattle. BMC Genomics 12: 318. 21679429 10.1186/1471-2164-12-318 PMC3146955

[16] International Chicken Polymorphism Map Consortium (2004) A genetic variation map for chicken with 2.8 million single-nucleotide polymorphisms. Nature 432: 717–722. 15592405 10.1038/nature03156 PMC2263125

[17] Rubin CJ, Zody MC, Eriksson J, Meadows JRS, Sherwood E, et al (2010) Whole-genome resequencing reveals loci under selection during chicken domestication. Nature 464: 587–591. 20220755 10.1038/nature08832

[18] Akey JM, Zhang G, Zhang K, Jin L, Shriver MD (2002) Interrogating a high-density SNP map for signatures of natural selection. Genome Res 12: 1805–1814. 12466284 10.1101/gr.631202 PMC187574

[19] Teshima KM, Coop G, Przeworski M (2006) How reliable are empirical genomic scans for selective sweeps? Genome Res 16: 702–712. 16687733 10.1101/gr.5105206 PMC1473181

[20] Kelley JL, Madeoy J, Calhoun JC, Swanson W, Akey JM (2006) Genomic signatures of positive selection in humans and the limits of outlier approaches. Genome Res 16: 980–989. 16825663 10.1101/gr.5157306 PMC1524870

[21] Williamson SH, Hubisz MJ, Clark AG, Payseur BA, Bustamante CD, et al (2007) Localizing recent adaptive evolution in the human genome. PLoS Genet 3: e90. 17542651 10.1371/journal.pgen.0030090 PMC1885279

[22] Kim Y, Stephan W (2002) Detecting a local signature of genetic hitchhiking along a recombining chromosome. Genetics 160: 765–777. 11861577 10.1093/genetics/160.2.765 PMC1461968

[23] Nielsen R, Williamson S, Kim Y, Hubisz MJ, Clark AG, et al (2005) Genomic scans for selective sweeps using SNP data. Genome Res 15: 1566–1575. 16251466 10.1101/gr.4252305 PMC1310644

[24] Pavlidis P, Jensen JD, Stephan W (2010) Searching for footprints of positive selection in whole-genome SNP data from non-equilibrium populations. Genetics 185: 907–922. 20407129 10.1534/genetics.110.116459 PMC2907208

[25] Weir BS, Cardon LR, Anderson AD, Nielsen DM, Hill WG (2005) Measures of human population structure show heterogeneity among genomic regions. Genome Res 15: 1468–1476. 16251456 10.1101/gr.4398405 PMC1310634

[26] Beccavin CB, Chevalier LA, Cogburn J, Simon, Duclos MJ (2001) Insulin-like growth factors and body growth in chickens divergently selected for high or low growth rate. J Endocrinol 168: 297–306. 11182767 10.1677/joe.0.1680297

[27] Zhou HJ, Mitchell AD, McMurtry JP, Ashwell CM, Lamont SJ (2005) Insulin-Like growth factor-I gene polymorphism associations with growth, body composition, skeleton integrity and metabolic traits in chickens. Poult Sci 84: 212–219. 15742956 10.1093/ps/84.2.212

[28] Cui JX, Du HL, Liang Y, Deng XM, Li N, et al (2006) Association of polymorphisms in the promoter region of chicken prolactin with egg production. Poult Sci 85: 26–31. 16493942 10.1093/ps/85.1.26

[29] Kuraku S, Kuratani S (2011) Genome-wide detection of gene extinction in early Mammalian evolution. Genome Biol Evol 3: 1449–62. 22094861 10.1093/gbe/evr120 PMC3296468

[30] Calenge F, Kaiser P, Vignal A, Beaumont C (2010) Genetic control of resistance to salmonellosis and to salmonella carrier-state in fowl: a review. Genet Sel Evol 42: 11. 20429884 10.1186/1297-9686-42-11 PMC2873309

[31] Li X, Yang Y, Zhou F, Zhang Y, Lu H, et al (2011) SLC11A1 (NRAMP1) polymorphisms and Tuberculosis susceptibility: Updated systematic review and Meta-analysis. PLoS ONE 6 (1) e15831. 21283567 10.1371/journal.pone.0015831 PMC3026788

[32] Twito T, Madeleine D, Perl-Treves R, Hillel J, Lavi U (2011) Comparative genome analysis with the human genome reveals chicken genes associated with fatness and body weight. Anim Genet 42: 642–649. 22035006 10.1111/j.1365-2052.2011.02191.x

[33] Nys Y, Mayel-Afshar S, Bouillon R, Van Balen H, Lawson DEM (1989) Increases in calbindin D 28K mRNA in the uterus of the domestic fowl induced by sexual maturity and shell formation. Gen Comp Endocrinol 76: 322–329. 2591722 10.1016/0016-6480(89)90164-0

[34] Striem S, Bar A (1991) Modulation of quail intestinal and egg shell gland Calbindin (M_r_ 28,000) gene expression by vitamin D_3_, 1,25-dihydroxyvitamin D_3_ and egg laying. Mol Cell Endocrinol 73: 169–177. 10.1016/0303-7207(91)90232-h 1646742

[35] Arazi H, Yoselewitz I, Malka Y, Kelner Y, Genin O, et al (2009) Osteopontin and Calbindin gene expression in the eggshell gland as related to eggshell abnormalities. Poult Sci 88 (3) 647–53. 19211537 10.3382/ps.2008-00387

[36] Kamata R, Shiraishi F, Izumi T, Takahashi S, Shimizu A, et al (2009) Mechanisms of estrogen-induced effects in avian reproduction caused by transovarian application of a xenoestrogen, diethylstilbestrol. Arch Arch Toxicol 83 (2) 161–71. 18597071 10.1007/s00204-008-0336-4

[37] Fernandez MS, Escobar C, Lavelin I, Pines M, Arias JL (2003) Localization of Osteopontin in oviduct tissue and eggshell during different stages of the avian egg laying cycle. J Struct Biol 143: 171–180. 14572472 10.1016/j.jsb.2003.08.007

[38] Hincke MT, Chien YC, Gerstenfeld LC, McKee MD (2008) Colloidal-gold immunocytochemical localization of osteopontin in avian eggshell gland and eggshell. J Histochem Cytochem 56: 467–476. 18256019 10.1369/jhc.2008.950576 PMC2324194

[39] Bennett AK, Hester PY, Spurlock DE (2006) Polymorphismsin Vitamin D receptor, osteopontin, insulin-like growth factor 1 and insulin, and their associations with bone, egg and growth traits in a layer – broiler cross in chickens. Anim Genet 37: 283–286. 16734694 10.1111/j.1365-2052.2006.01439.x

[40] Hellgren O, Ekblom R (2010) Evolution of a cluster of innate immune genes (beta-defensins) along the ancestral lines of chicken and zebra finch. Immunome Research 6: 3. 20359324 10.1186/1745-7580-6-3 PMC3161384

[41] Hervé-Grépinet V, Réhault-Godbert S, Labas V, Magallon T, Derache C, et al (2010) Purification and Characterization of Avian ß-Defensin 11, an Antimicrobial Peptide of the Hen Egg. Antimicrob Agents Chemother 54 (10) 4401–4409. 20625158 10.1128/AAC.00204-10 PMC2944589

[42] Mulsant P, Lecerf F, Fabre S, Schibler L, Monget P, et al (2001) Mutation in bone morphogenetic protein receptor- IB is associated with increased ovulation rate in Booroola Merino ewes. Proc Natl Acad Sci 98: 5104–109. 11320249 10.1073/pnas.091577598 PMC33171

[43] Souza CJH, MacDougall C, Campbell BK, McNeilly AS, Baird DT (2001) The Booroola (FecB) phenotype is associated with a mutation in the bone morphogenetic receptor type 1B (BMPRIB) gene. J Endocrinol 169: 1–6. 11312159 10.1677/joe.0.169r001

[44] Sambrook J, Russell DW (2001) Molecular Cloning: A Laboratory Manual. Cold Spring Harbor Laboratory, New York, USA.

[45] Li H, Handsaker B, Wysoker A, Fennell T, Ruan J, et al (2009) The Sequence alignment/map (SAM) format and SAMtools. Bioinformatics 25: 2078–2079. 19505943 10.1093/bioinformatics/btp352 PMC2723002

[46] Churchill GA, Doerge RW (1994) Empirical threshold values for quantitative trait mapping. Genetics 138 (3) 963–971. 7851788 10.1093/genetics/138.3.963 PMC1206241

[47] Ewing G, Hermisson J (2010) MSMS: a coalescent simulation program including recombination, demographic structure and selection at a single locus. Bioinformatics 26: 2064–2065. 20591904 10.1093/bioinformatics/btq322 PMC2916717

