# Supplementary figures and images for: A High Resolution Genome-Wide Scan for Significant Selective Sweeps: An Application to Pooled Sequence Data in Laying Chickens
Source: PLoS One. 2012 Nov 29;7(11):e49525. doi: 10.1371/journal.pone.0049525 (PMC3510216; doi:10.1371/journal.pone.0049525)

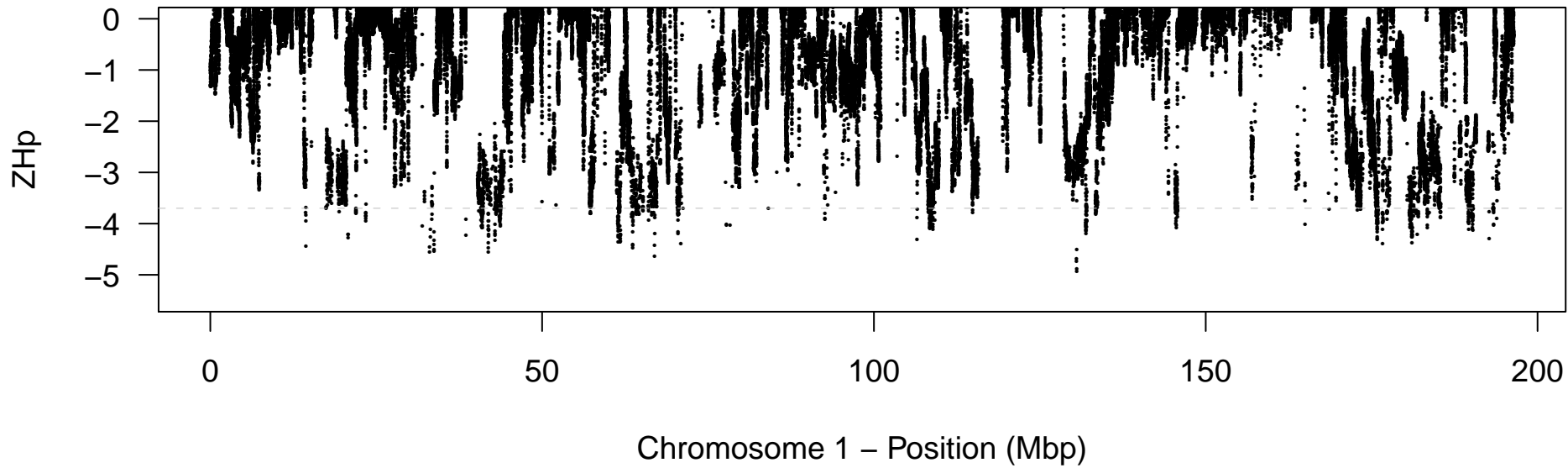

Supplement: Figure S1 — Chromosome wide distribution of variability measured in overlapping windows of 40 k. (PDF) [file pone.0049525.s001.pdf]

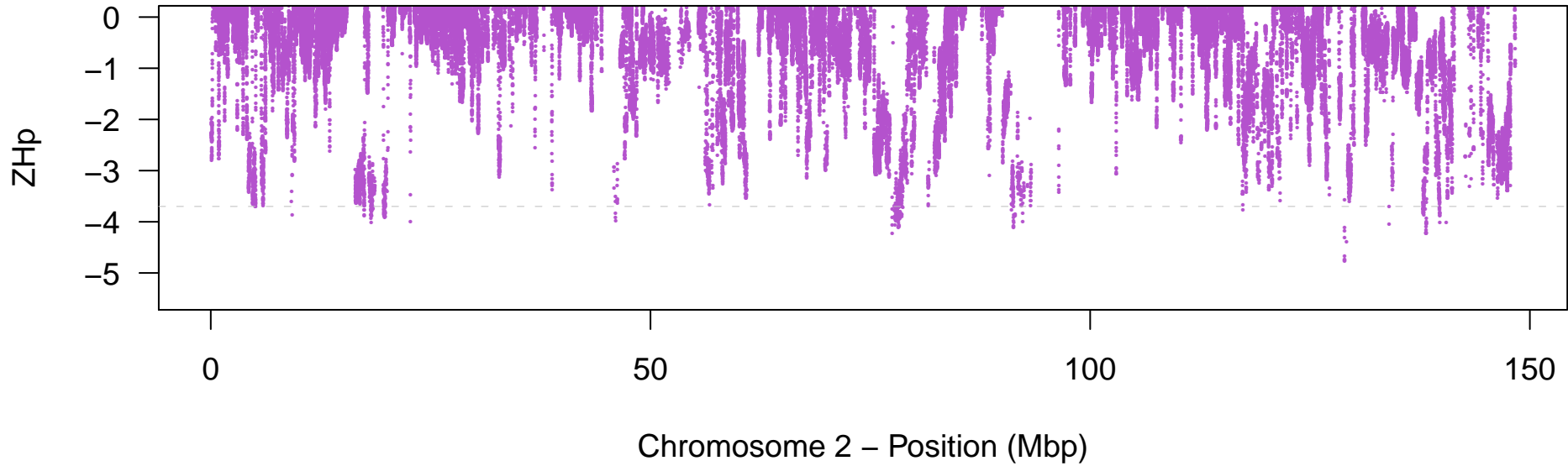

Supplement: Figure S2 — Chromosome wide distribution of variability measured in overlapping windows of 40 k. (PDF) [file pone.0049525.s002.pdf]

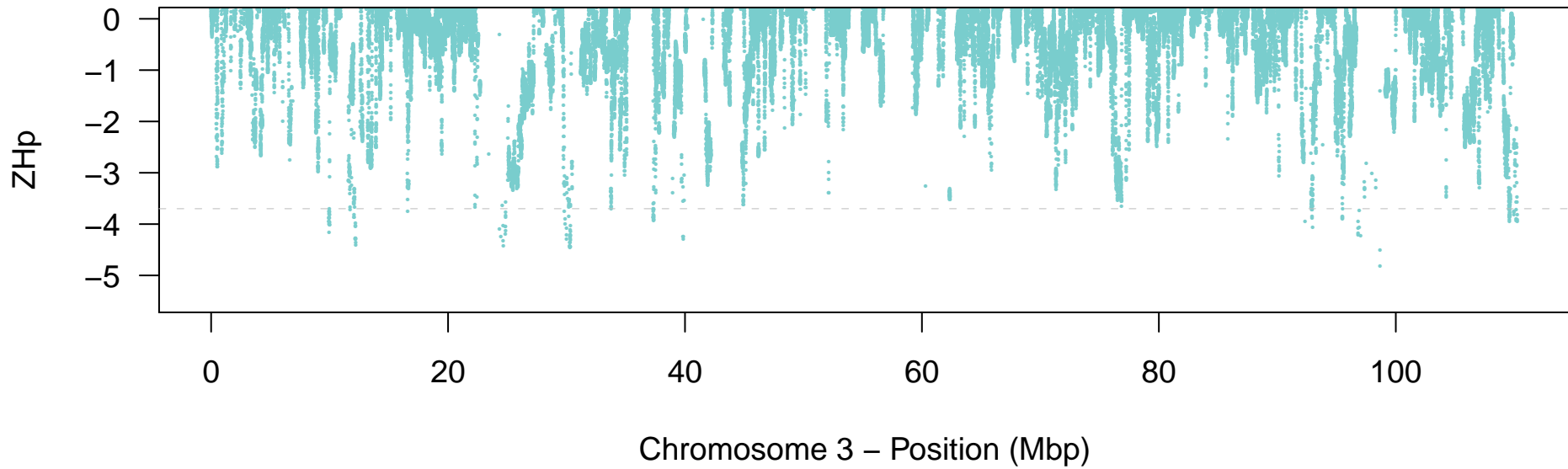

Supplement: Figure S3 — Chromosome wide distribution of variability measured in overlapping windows of 40 k. (PDF) [file pone.0049525.s003.pdf]

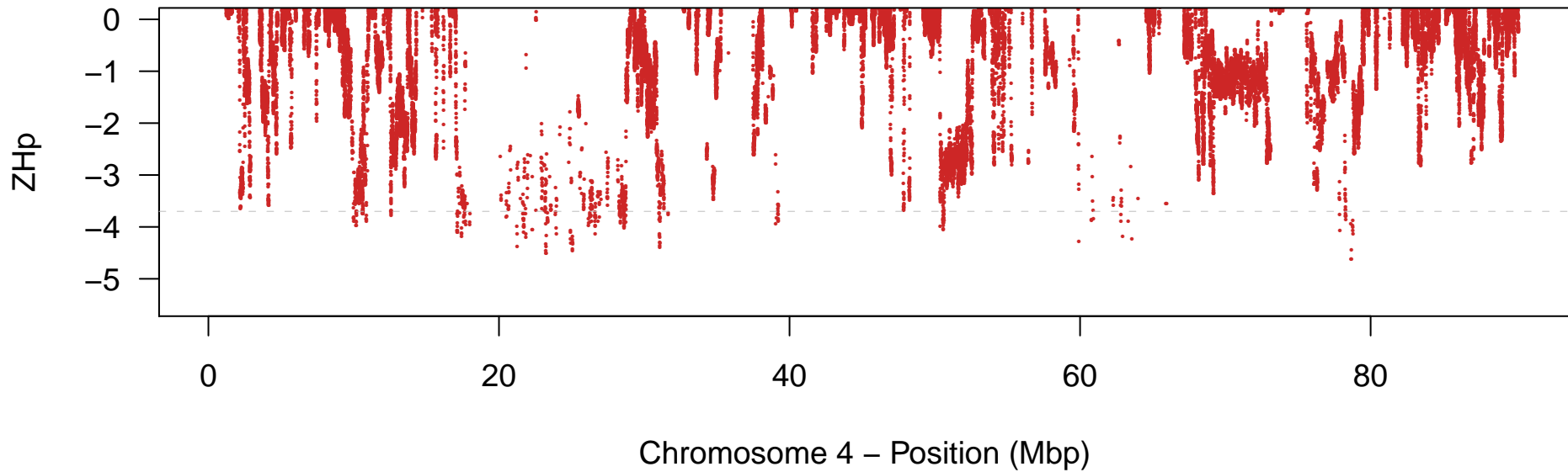

Supplement: Figure S4 — Chromosome wide distribution of variability measured in overlapping windows of 40 k. (PDF) [file pone.0049525.s004.pdf]

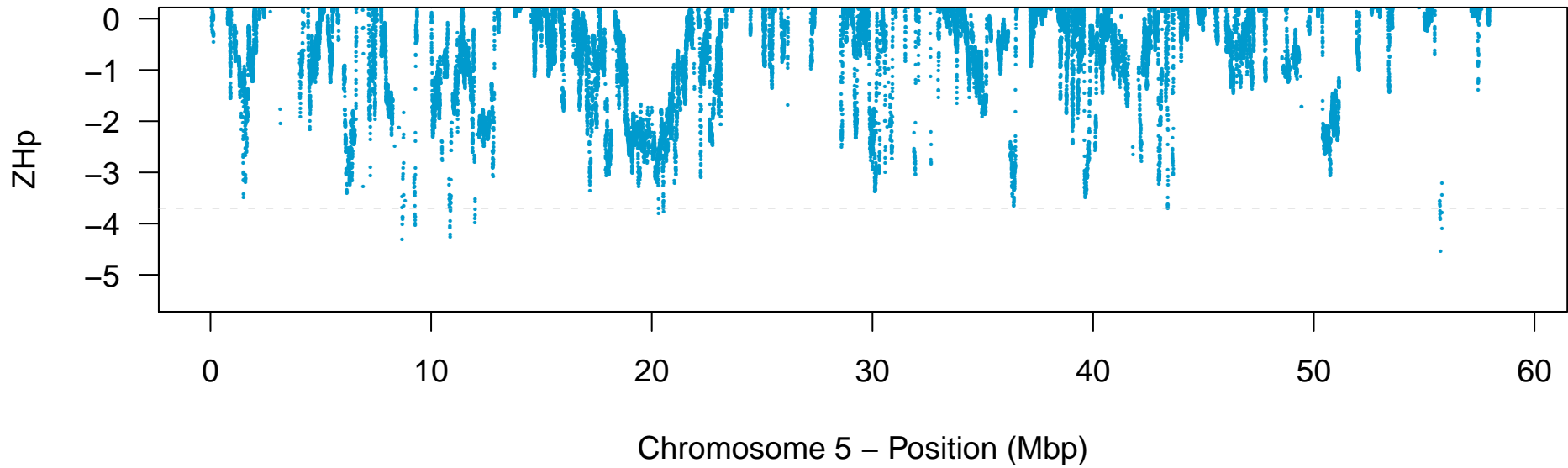

Supplement: Figure S5 — Chromosome wide distribution of variability measured in overlapping windows of 40 k. (PDF) [file pone.0049525.s005.pdf]

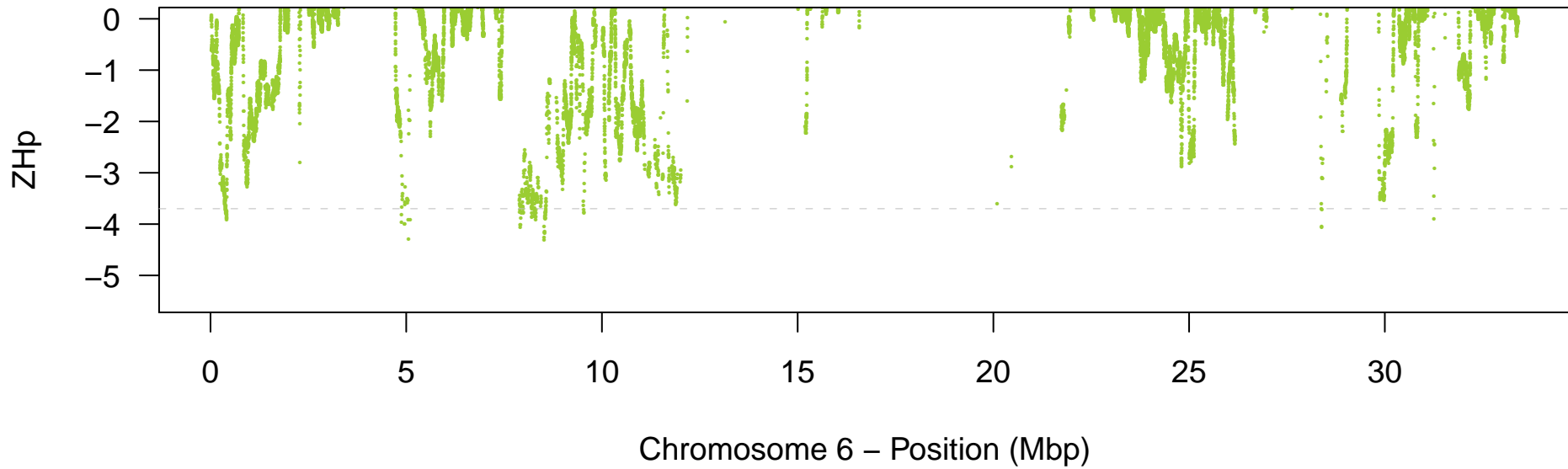

Supplement: Figure S6 — Chromosome wide distribution of variability measured in overlapping windows of 40 k. (PDF) [file pone.0049525.s006.pdf]

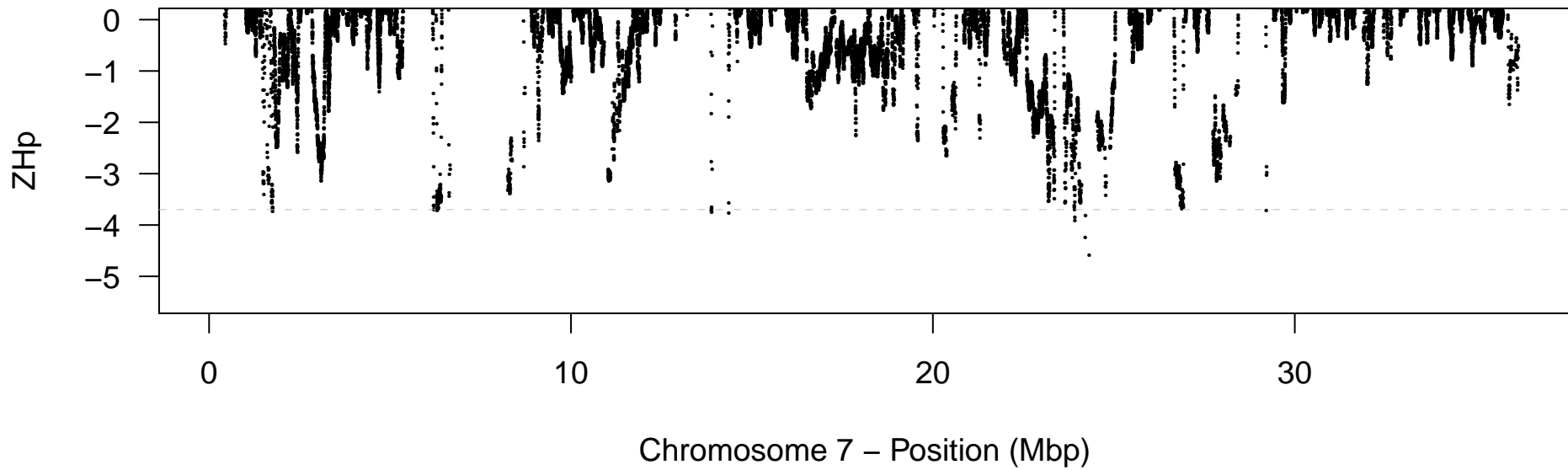

Supplement: Figure S7 — Chromosome wide distribution of variability measured in overlapping windows of 40 k. (PDF) [file pone.0049525.s007.pdf]

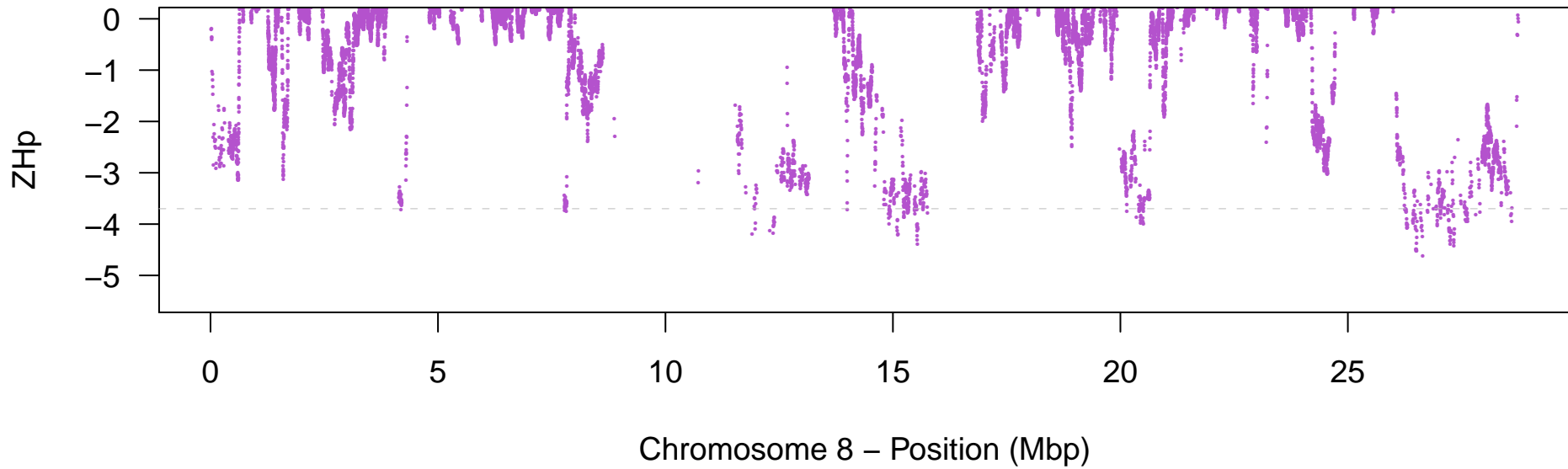

Supplement: Figure S8 — Chromosome wide distribution of variability measured in overlapping windows of 40 k. (PDF) [file pone.0049525.s008.pdf]

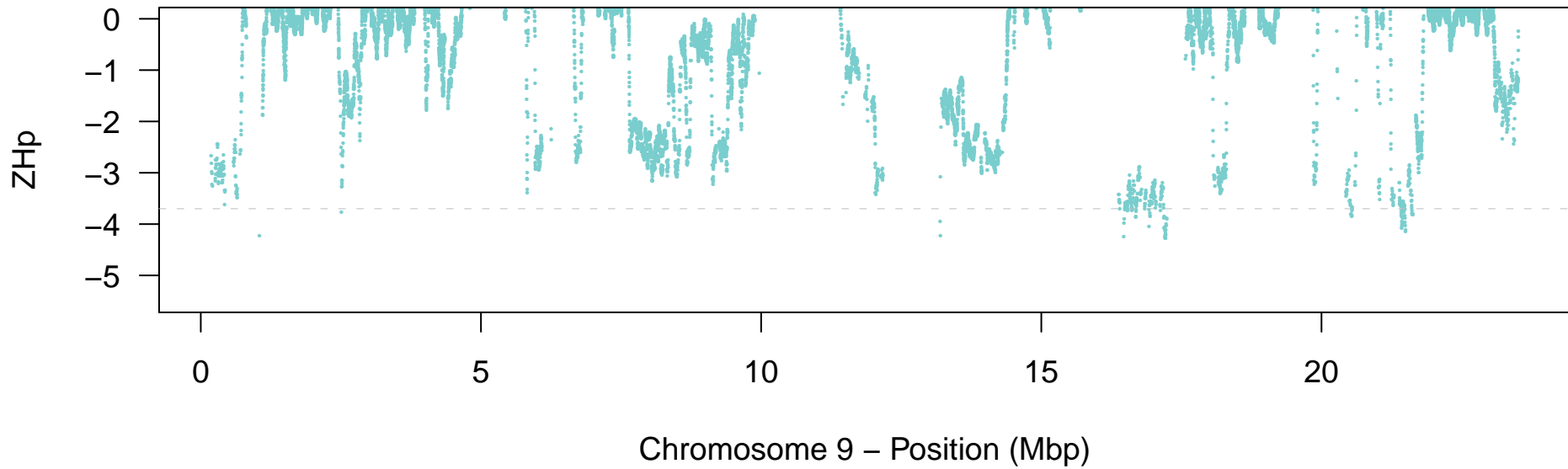

Supplement: Figure S9 — Chromosome wide distribution of variability measured in overlapping windows of 40 k. (PDF) [file pone.0049525.s009.pdf]

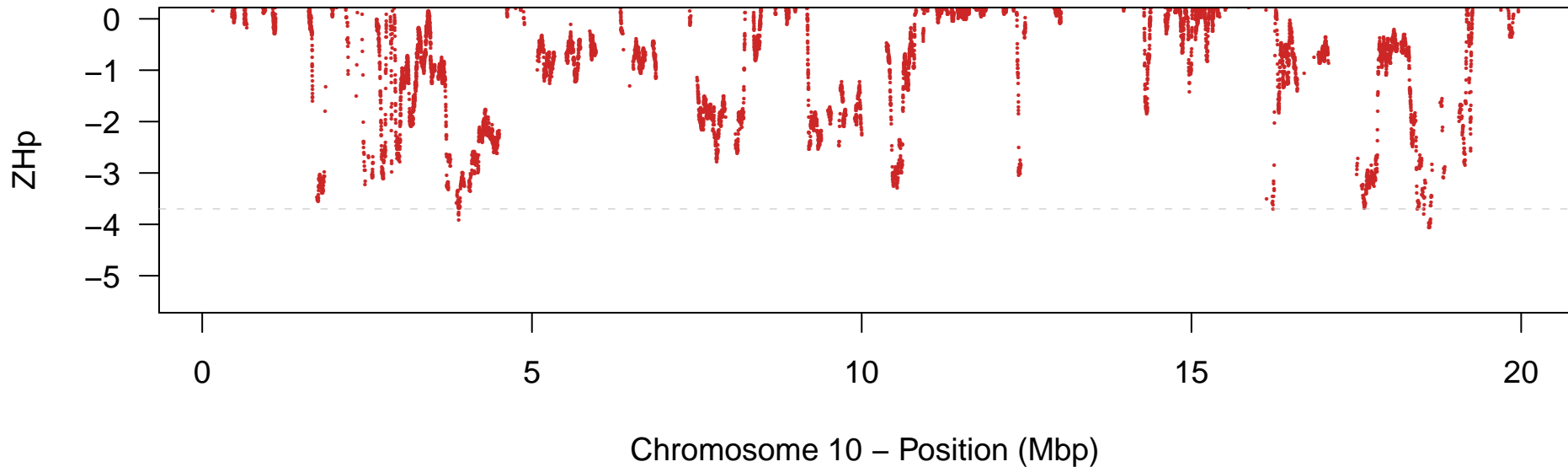

Supplement: Figure S10 — Chromosome wide distribution of variability measured in overlapping windows of 40 k. (PDF) [file pone.0049525.s010.pdf]

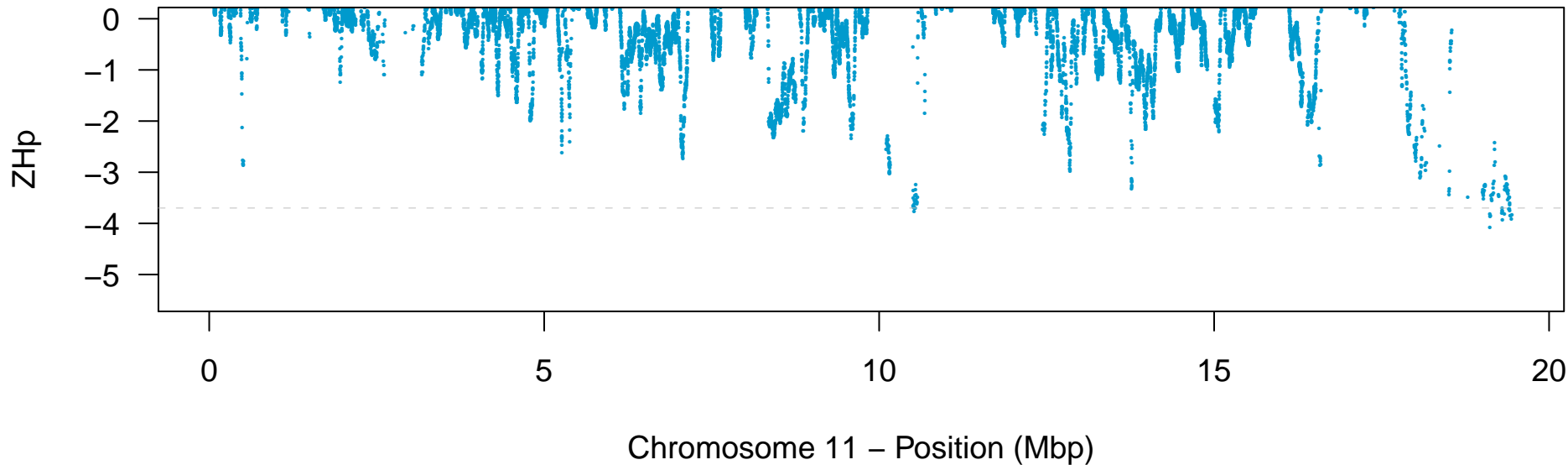

Supplement: Figure S11 — Chromosome wide distribution of variability measured in overlapping windows of 40 k. (PDF) [file pone.0049525.s011.pdf]

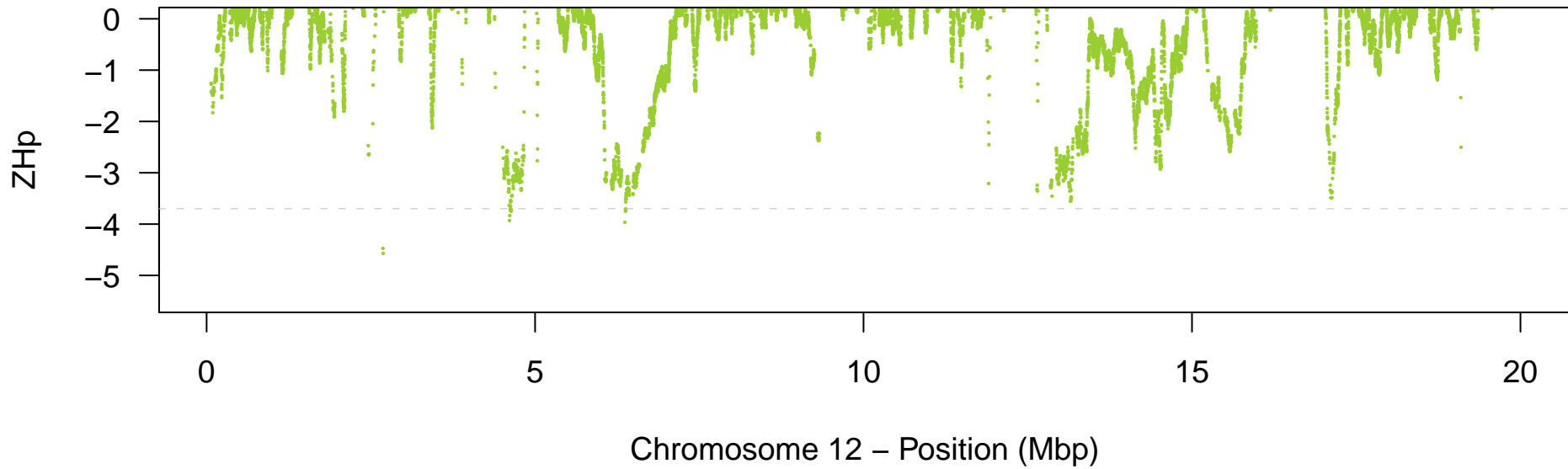

Supplement: Figure S12 — Chromosome wide distribution of variability measured in overlapping windows of 40 k. (PDF) [file pone.0049525.s012.pdf]

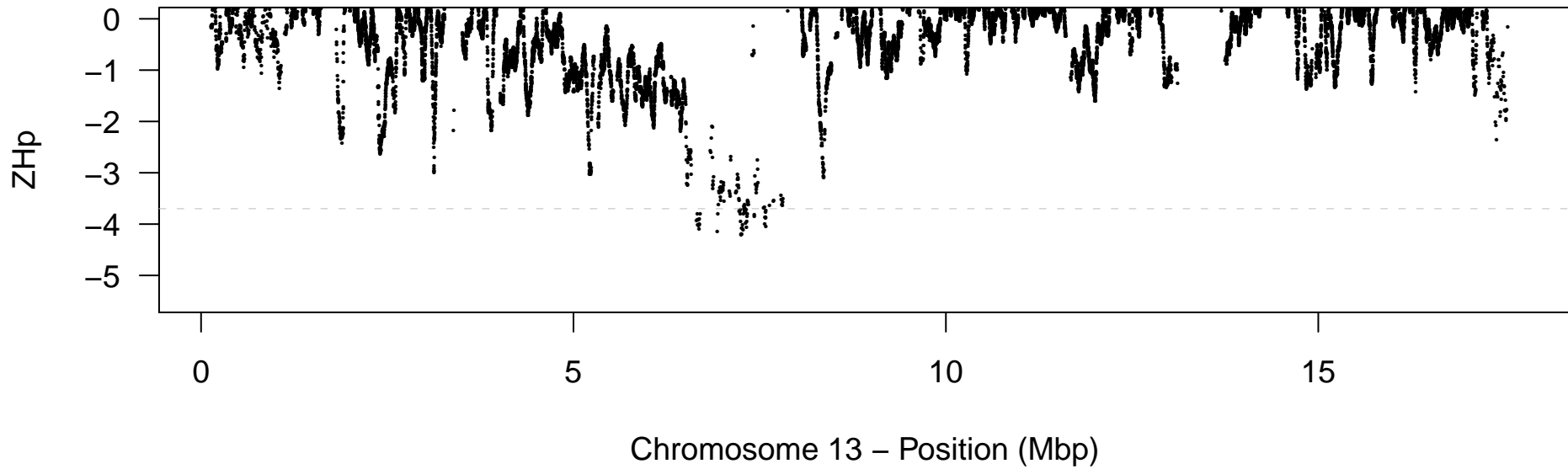

Supplement: Figure S13 — Chromosome wide distribution of variability measured in overlapping windows of 40 k. (PDF) [file pone.0049525.s013.pdf]

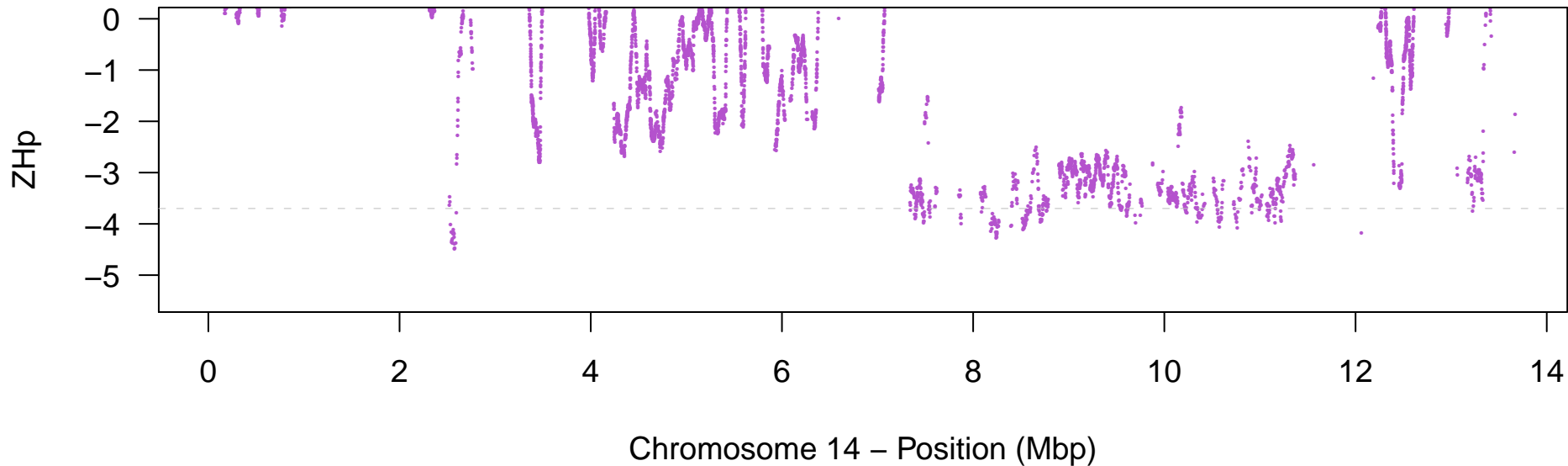

Supplement: Figure S14 — Chromosome wide distribution of variability measured in overlapping windows of 40 k. (PDF) [file pone.0049525.s014.pdf]

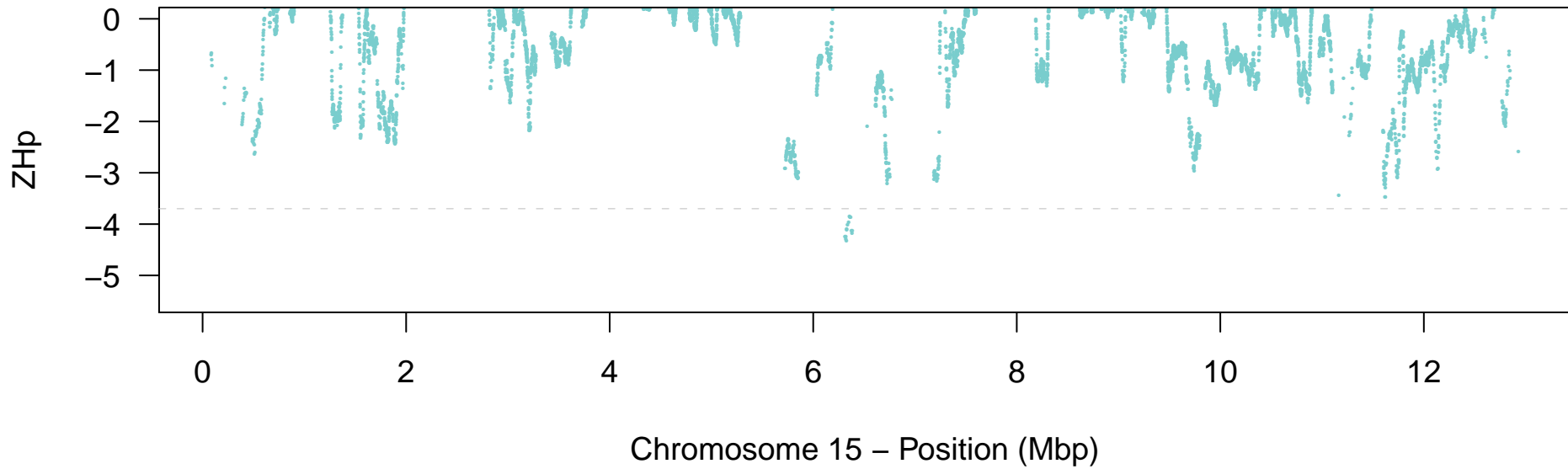

Supplement: Figure S15 — Chromosome wide distribution of variability measured in overlapping windows of 40 k. (PDF) [file pone.0049525.s015.pdf]

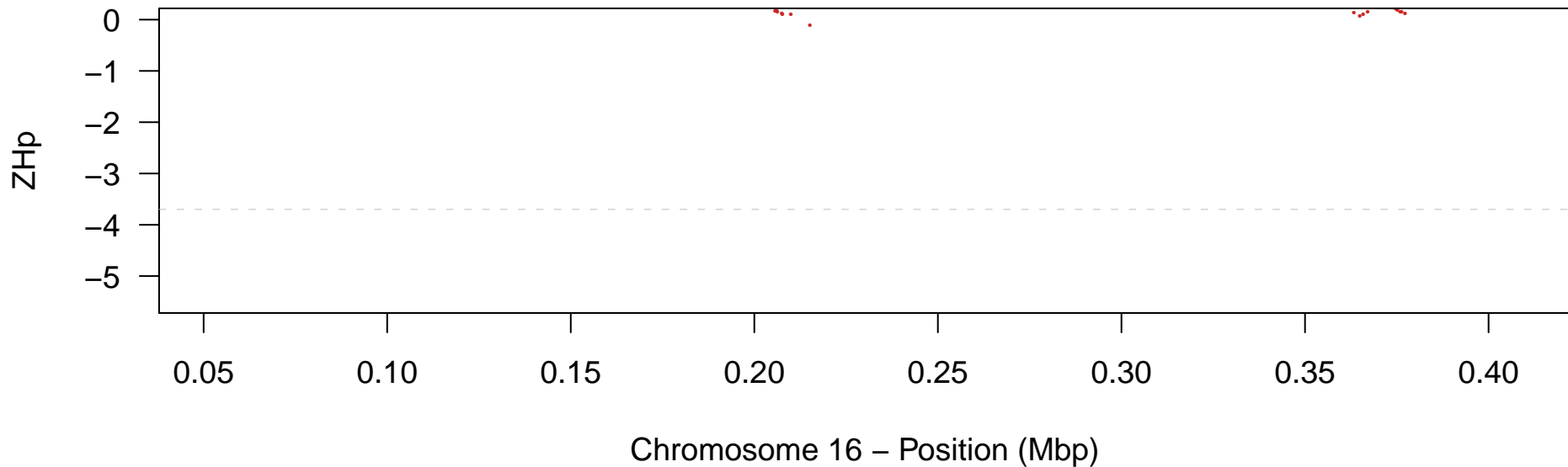

Supplement: Figure S16 — Chromosome wide distribution of variability measured in overlapping windows of 40 k. (PDF) [file pone.0049525.s016.pdf]

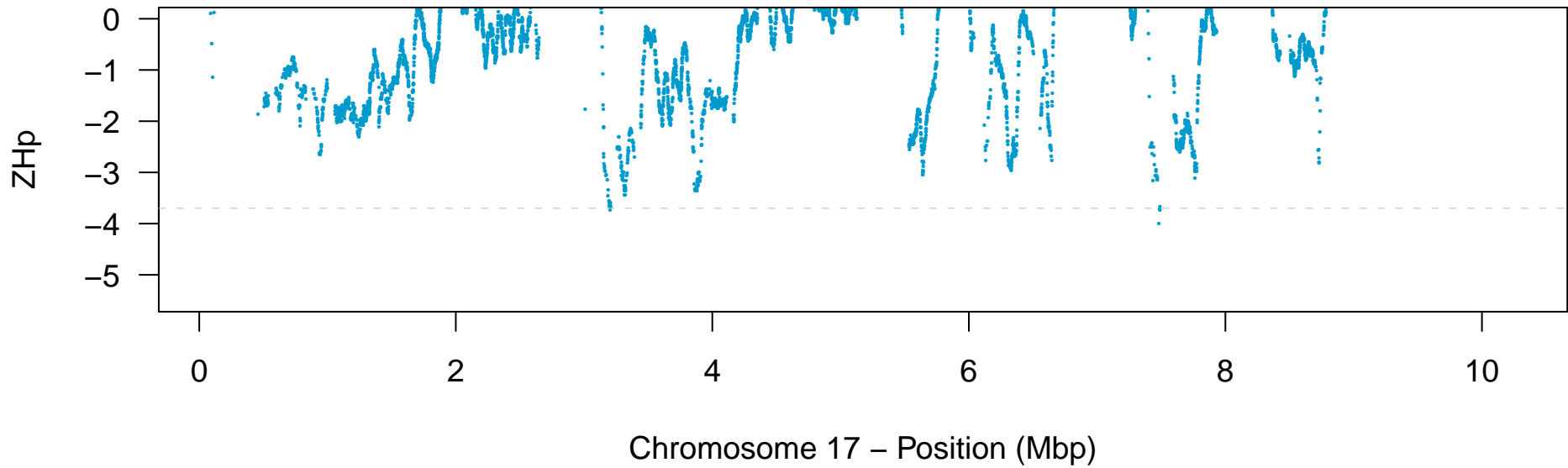

Supplement: Figure S17 — Chromosome wide distribution of variability measured in overlapping windows of 40 k. (PDF) [file pone.0049525.s017.pdf]

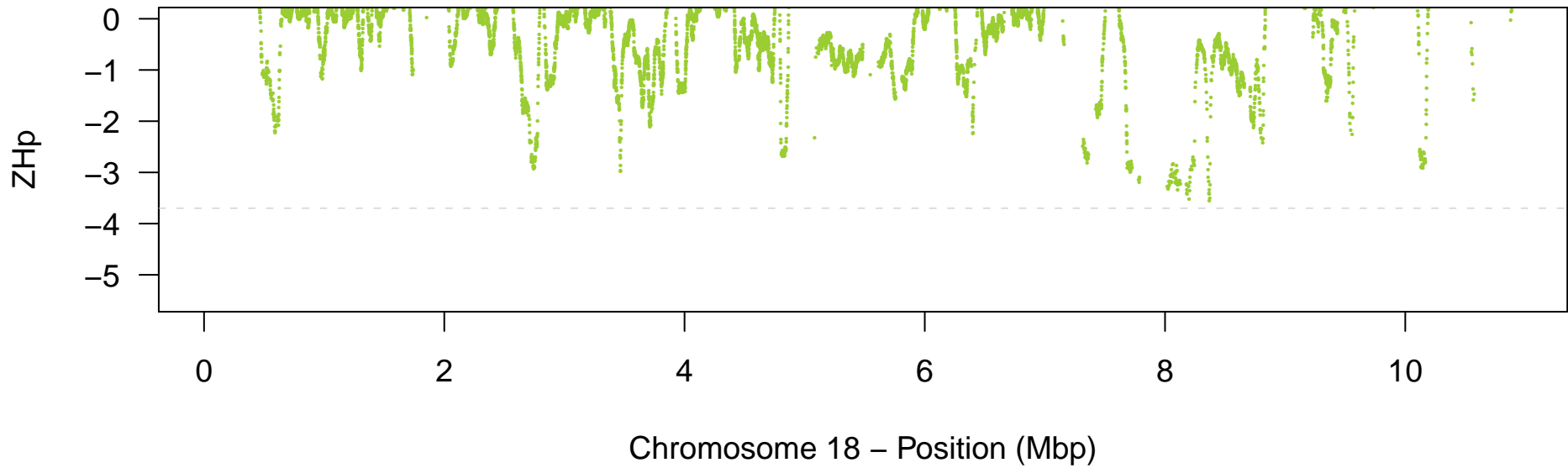

Supplement: Figure S18 — Chromosome wide distribution of variability measured in overlapping windows of 40 k. (PDF) [file pone.0049525.s018.pdf]

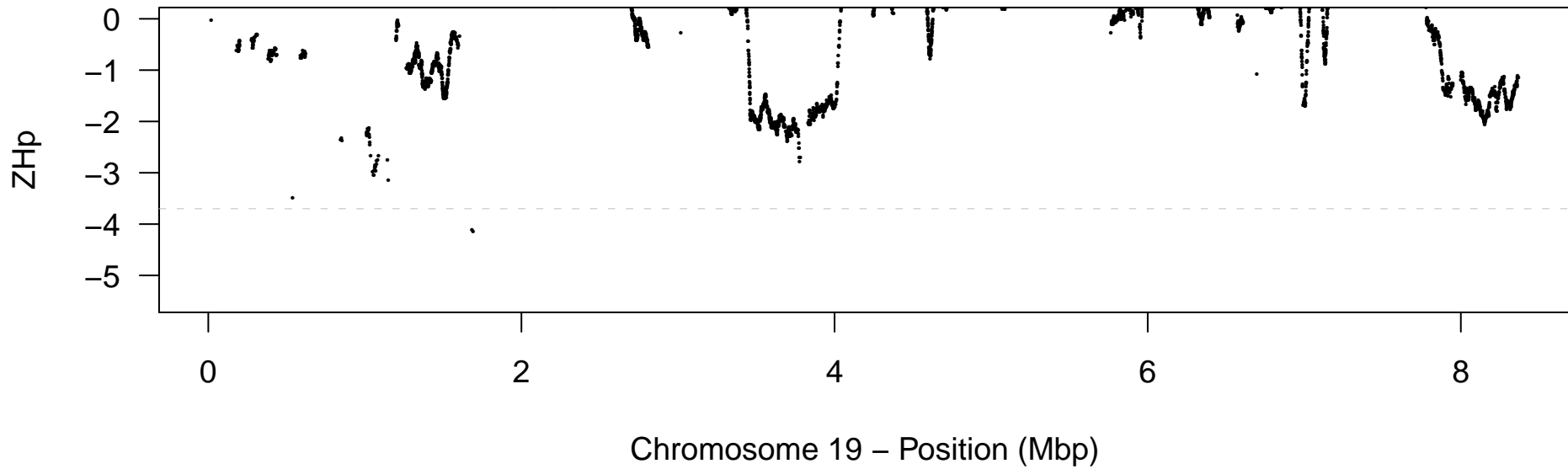

Supplement: Figure S19 — Chromosome wide distribution of variability measured in overlapping windows of 40 k. (PDF) [file pone.0049525.s019.pdf]

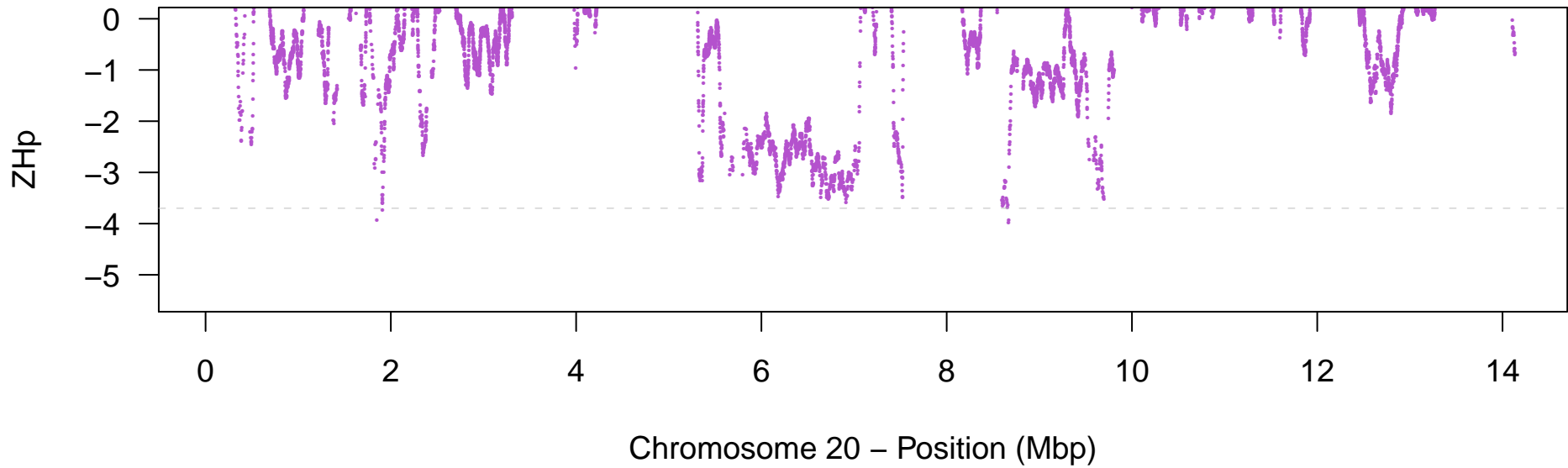

Supplement: Figure S20 — Chromosome wide distribution of variability measured in overlapping windows of 40 k. (PDF) [file pone.0049525.s020.pdf]

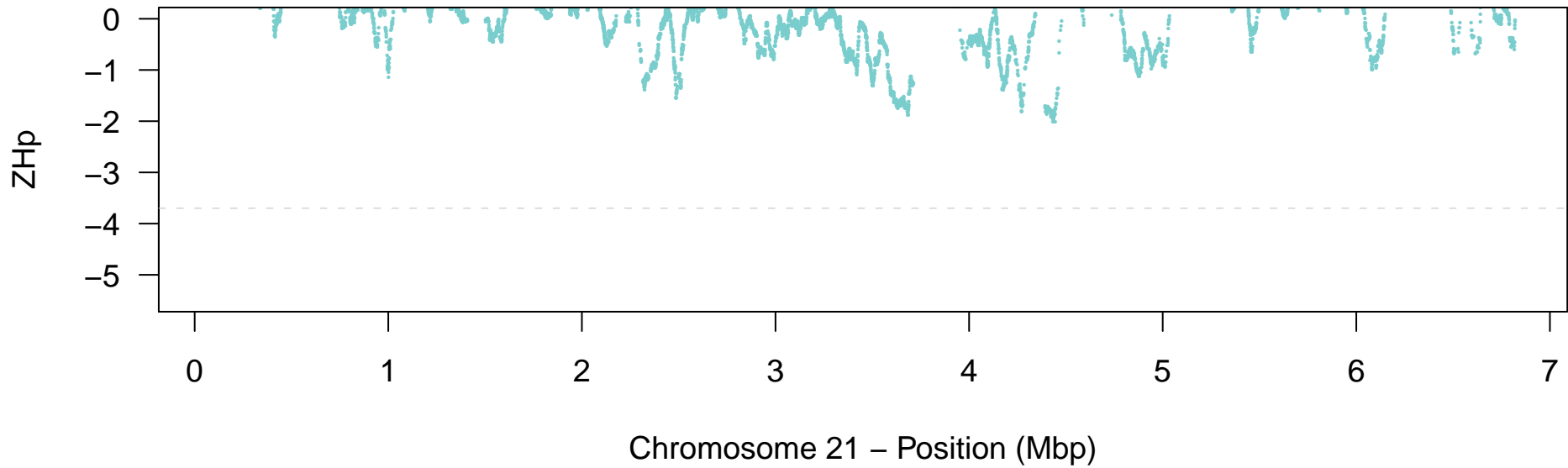

Supplement: Figure S21 — Chromosome wide distribution of variability measured in overlapping windows of 40 k. (PDF) [file pone.0049525.s021.pdf]

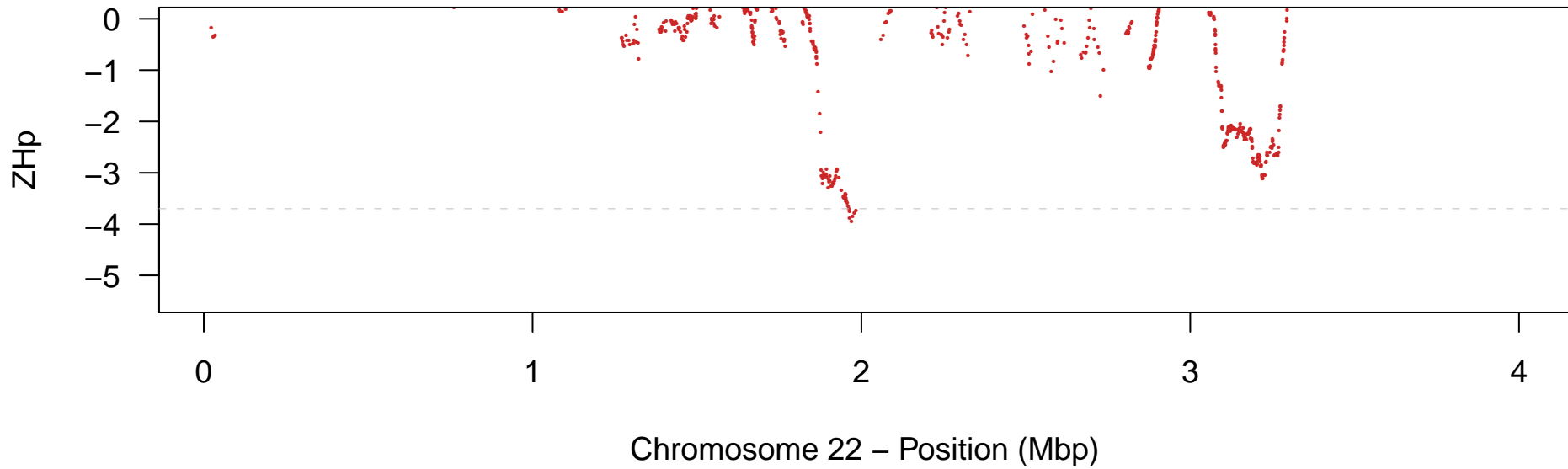

Supplement: Figure S22 — Chromosome wide distribution of variability measured in overlapping windows of 40 k. (PDF) [file pone.0049525.s022.pdf]

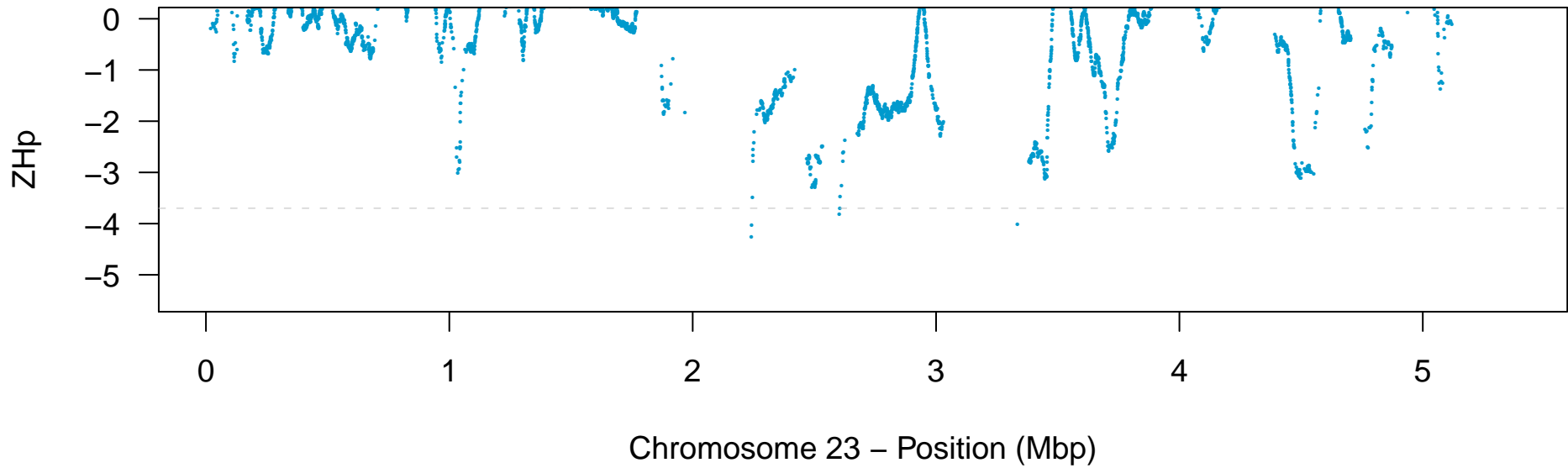

Supplement: Figure S23 — Chromosome wide distribution of variability measured in overlapping windows of 40 k. (PDF) [file pone.0049525.s023.pdf]

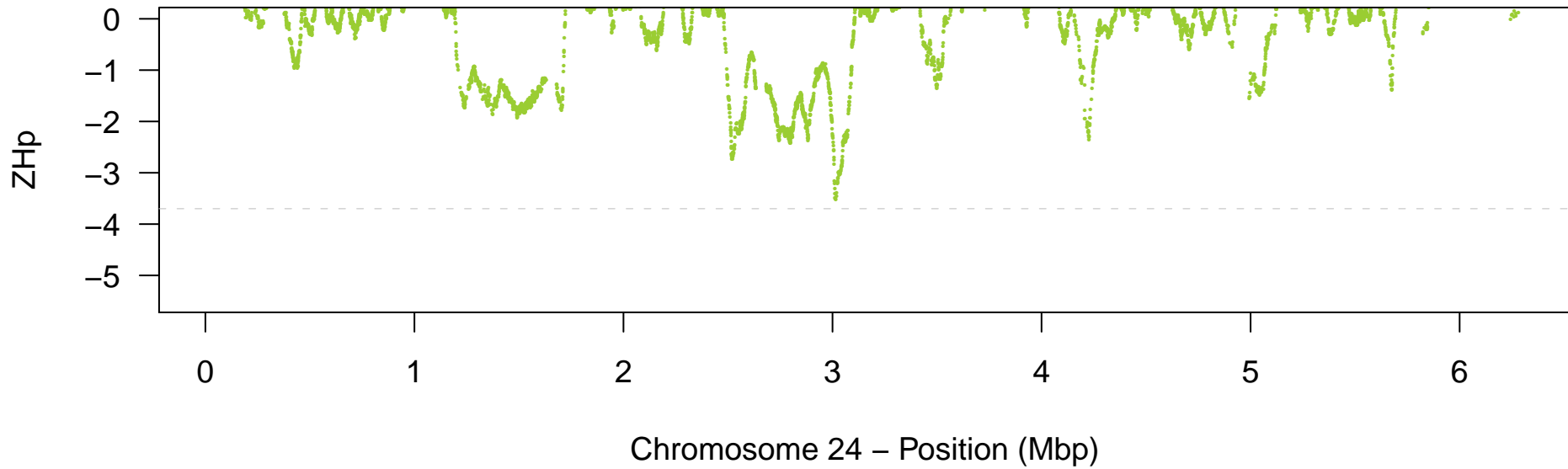

Supplement: Figure S24 — Chromosome wide distribution of variability measured in overlapping windows of 40 k. (PDF) [file pone.0049525.s024.pdf]

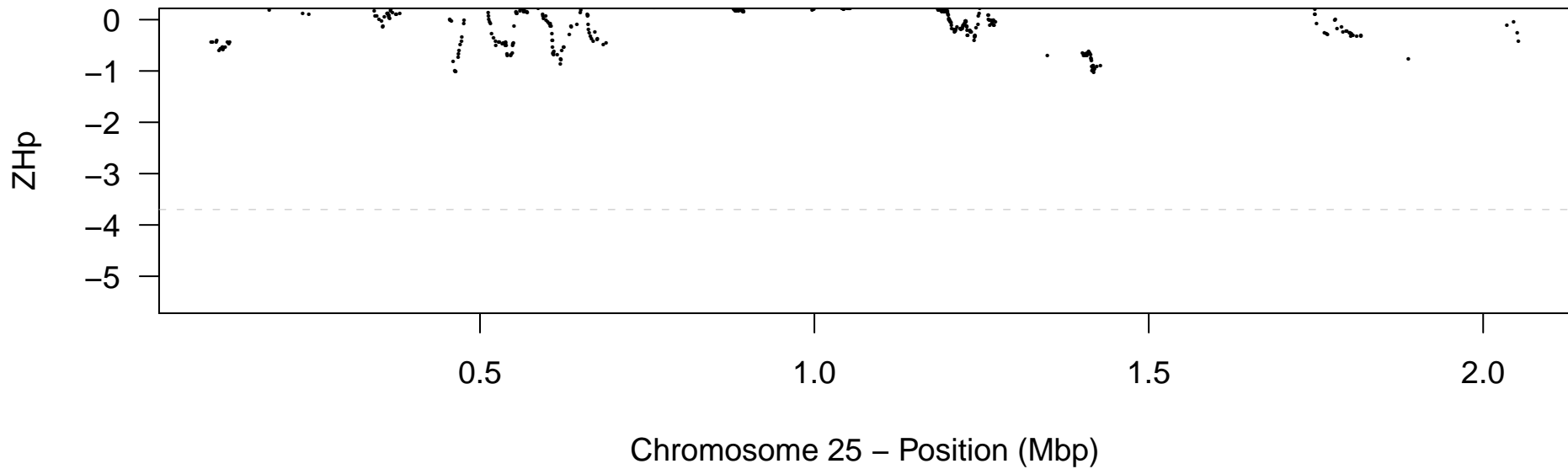

Supplement: Figure S25 — Chromosome wide distribution of variability measured in overlapping windows of 40 k. (PDF) [file pone.0049525.s025.pdf]

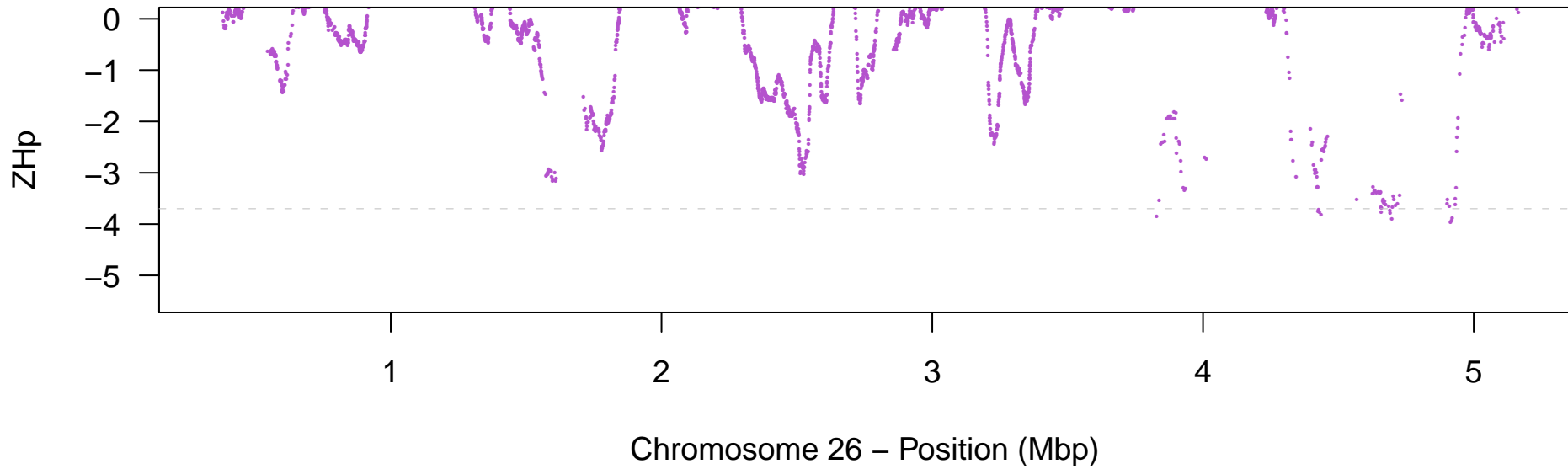

Supplement: Figure S26 — Chromosome wide distribution of variability measured in overlapping windows of 40 k. (PDF) [file pone.0049525.s026.pdf]

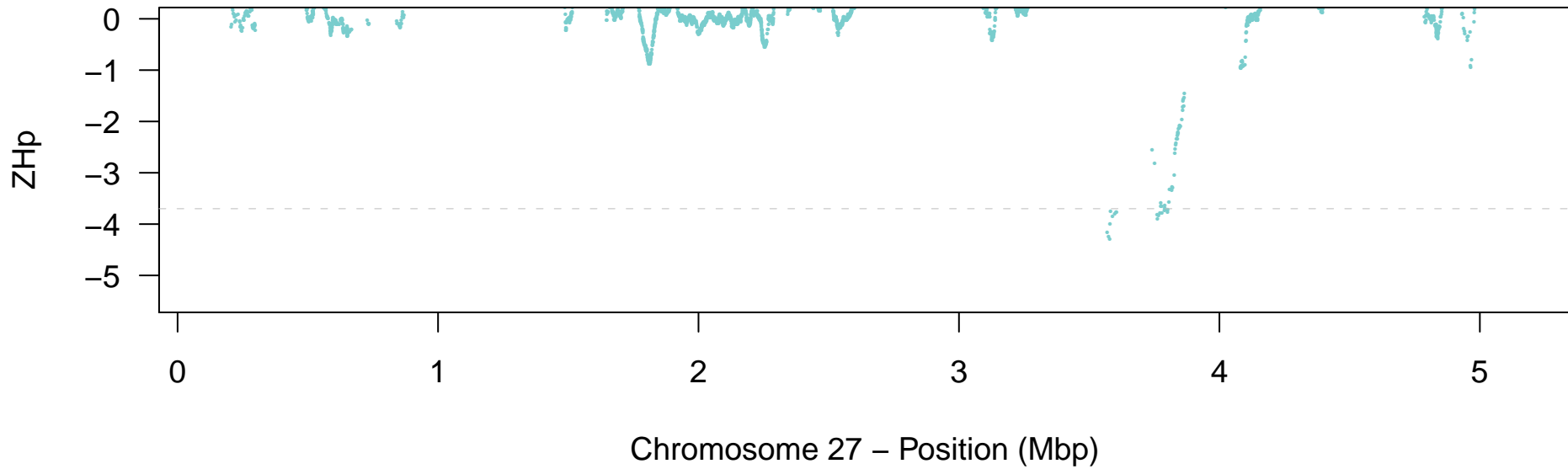

Supplement: Figure S27 — Chromosome wide distribution of variability measured in overlapping windows of 40 k. (PDF) [file pone.0049525.s027.pdf]

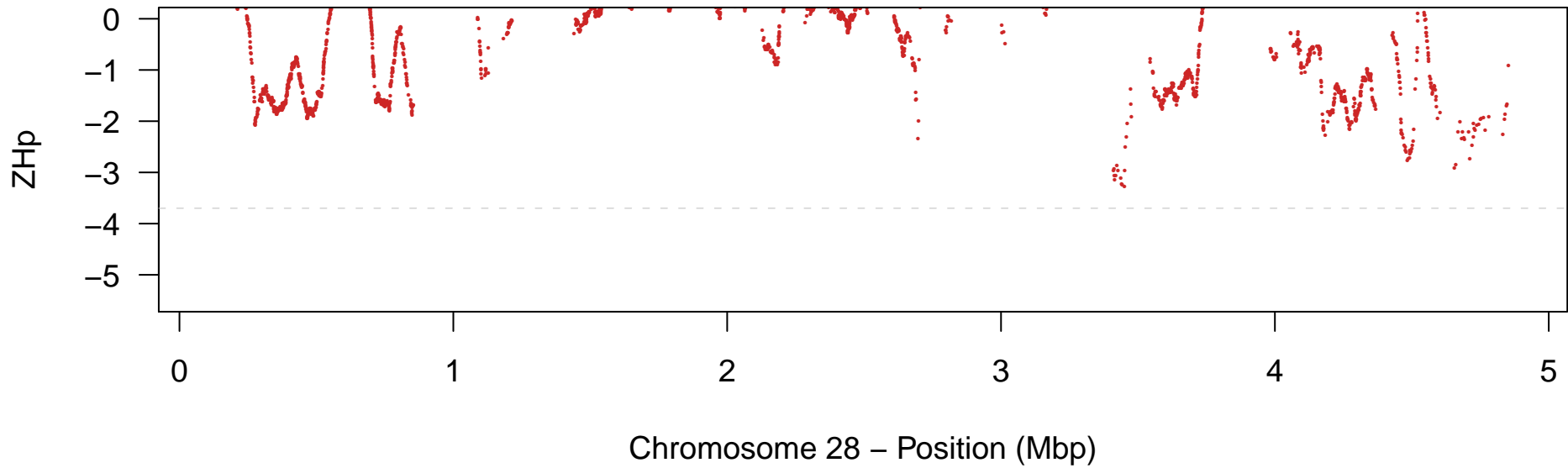

Supplement: Figure S28 — Chromosome wide distribution of variability measured in overlapping windows of 40 k. (PDF) [file pone.0049525.s028.pdf]

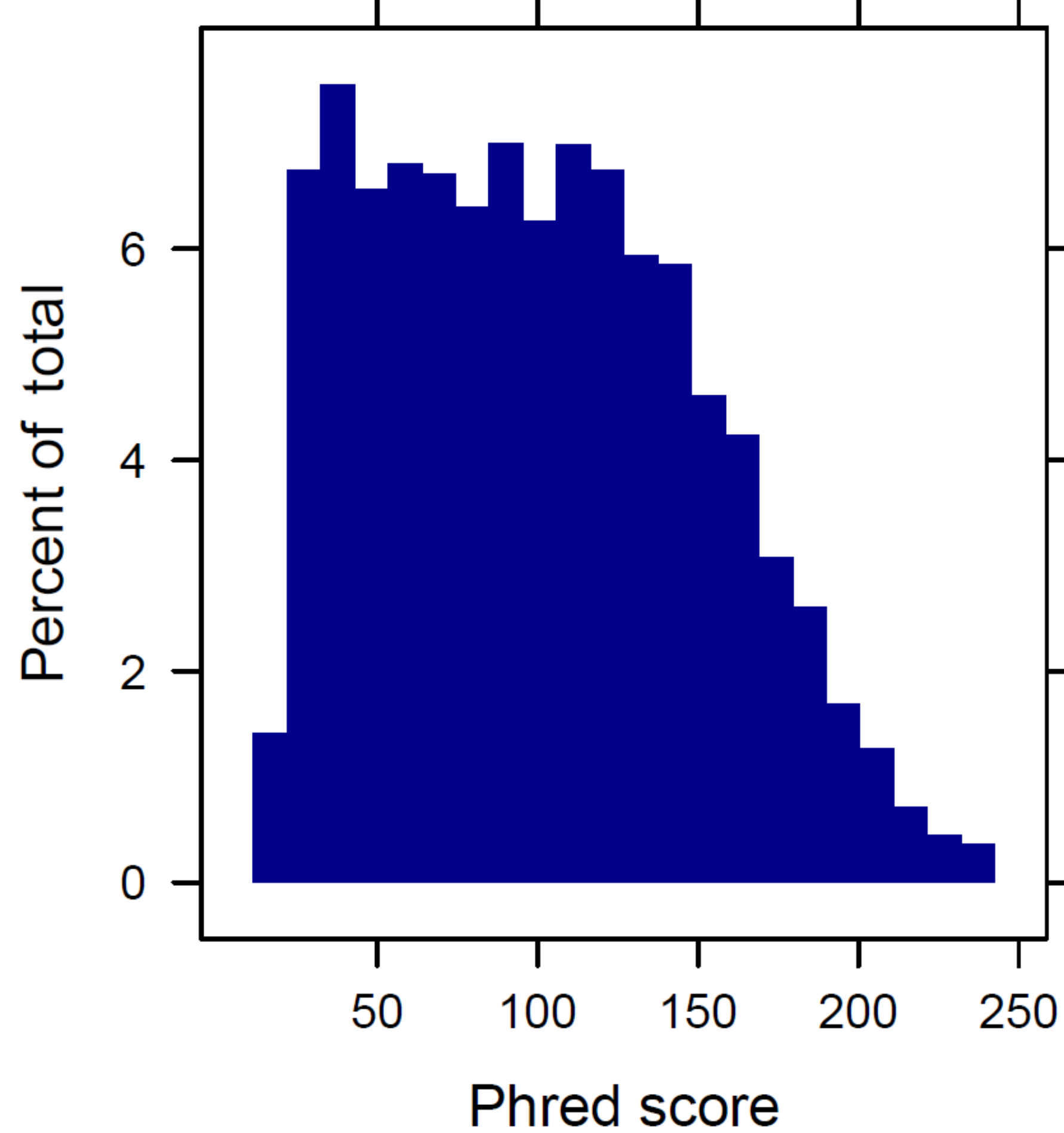

Supplement: Figure S29 — Phred quality score distribution. (PDF) [file pone.0049525.s029.pdf]

Percent of total

15

10

5

0

20

30

40

50

SNP calling read

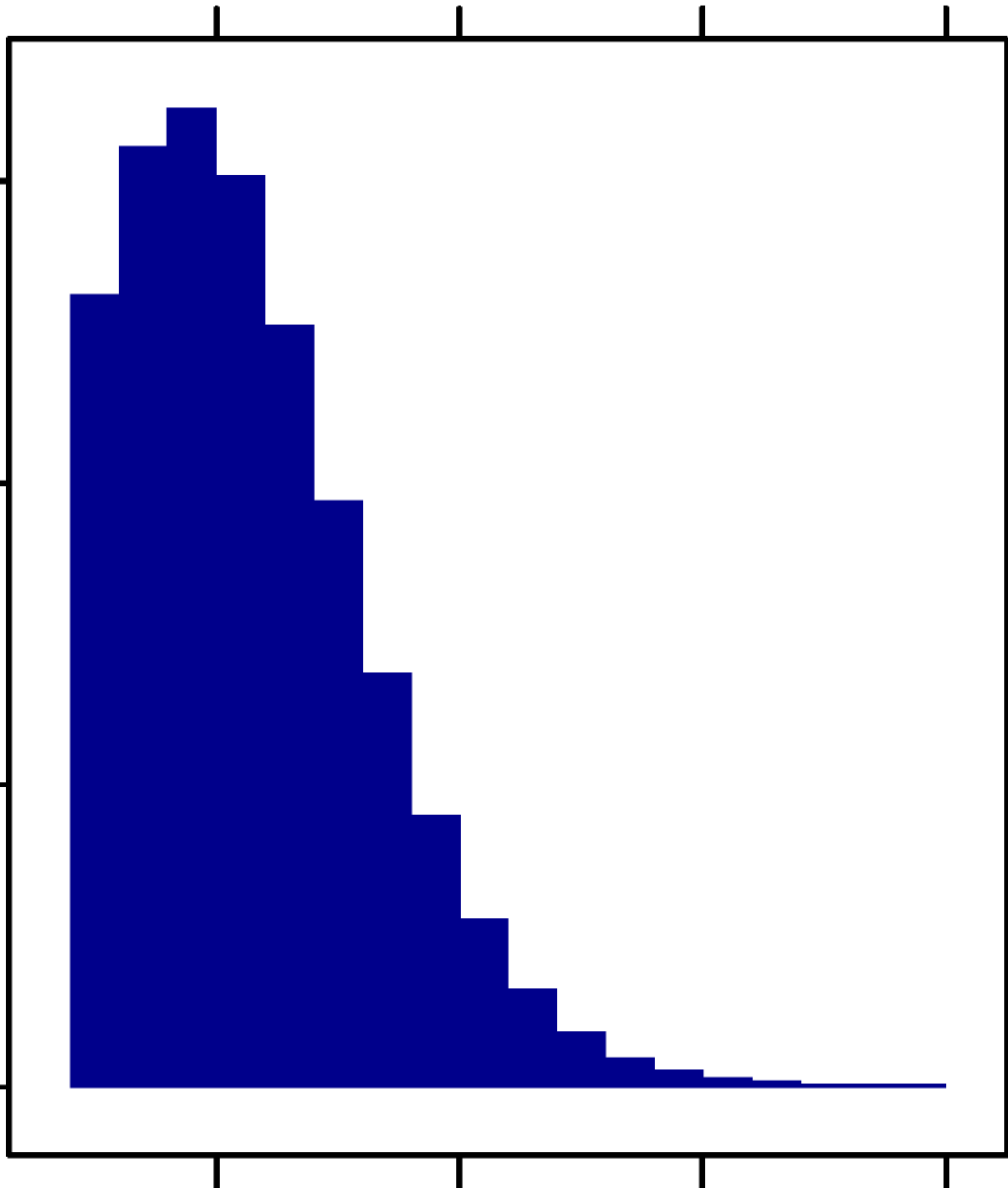

Supplement: Figure S30 — Distribution of the calling read of SNPs in final data set. (PDF) [file pone.0049525.s030.pdf]

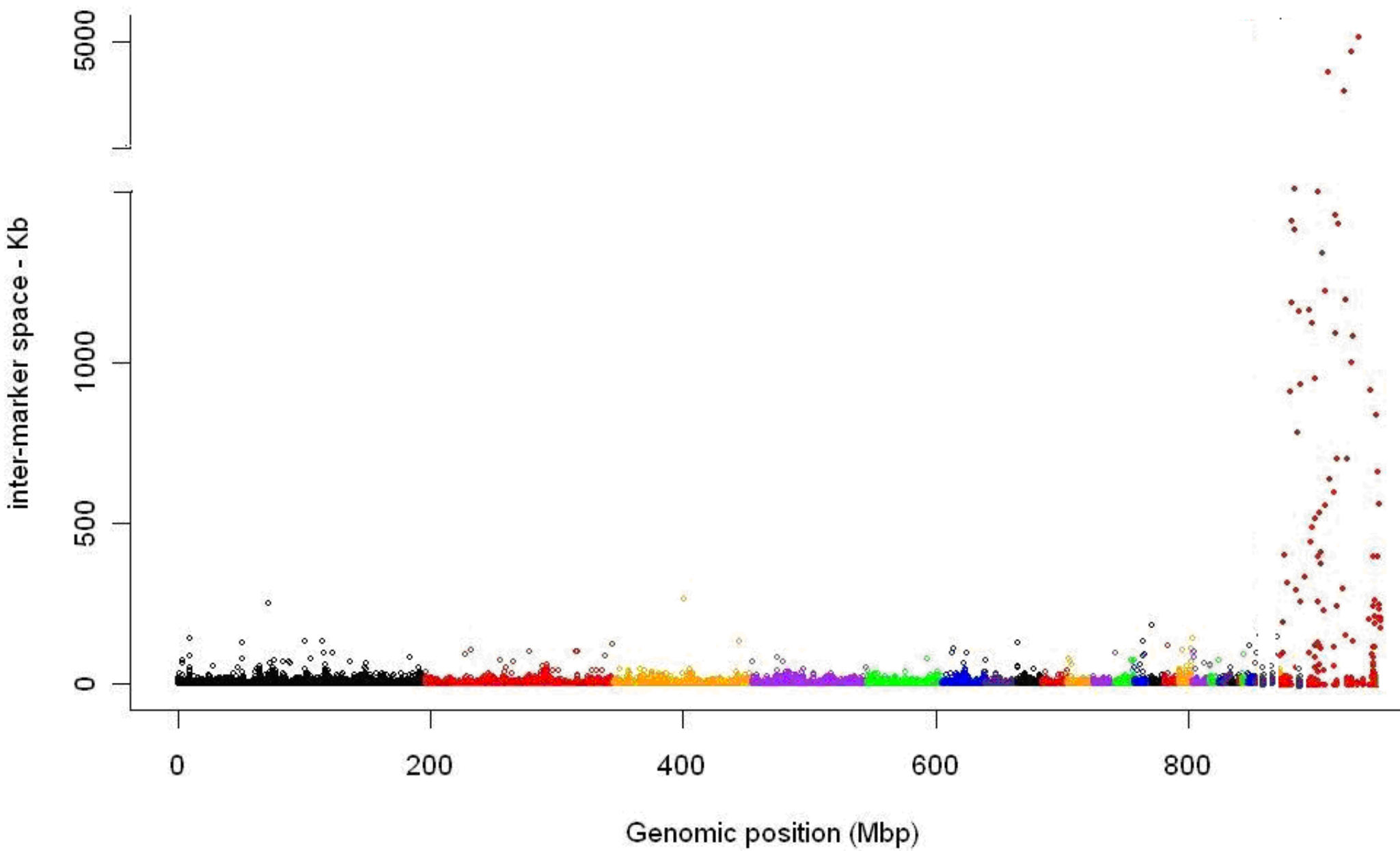

Supplement: Figure S31 — A genome wide inter marker distance between neighboring markers before data cleaning. (PDF) [file pone.0049525.s031.pdf]

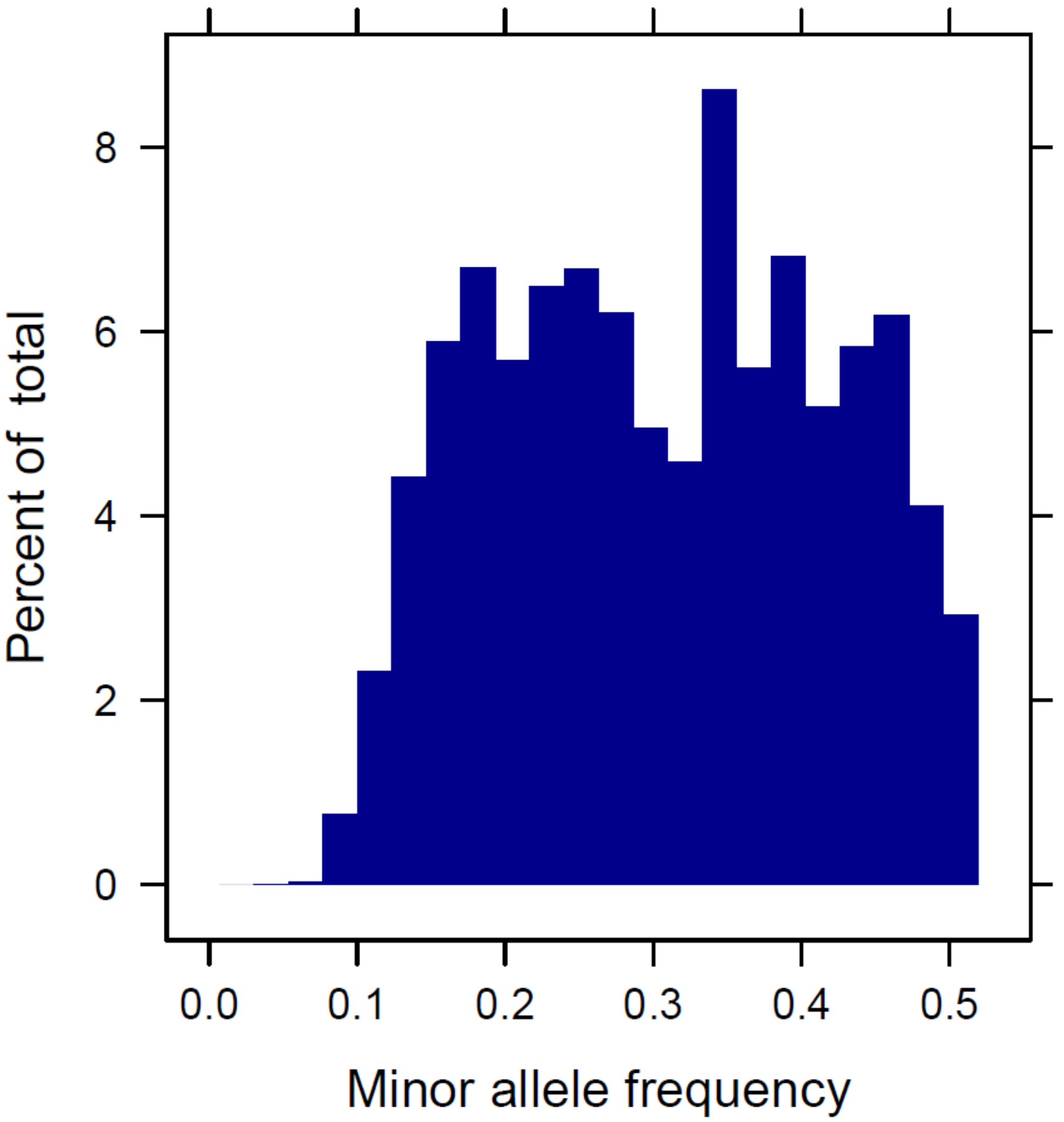

Supplement: Figure S32 — Frequency distribution of minor allele frequencies involved in final analysis. (PDF) [file pone.0049525.s032.pdf]
