# Supplementary material for: A High Resolution Genome-Wide Scan for Significant Selective Sweeps: An Application to Pooled Sequence Data in Laying Chickens
Source: PLoS One. 2012 Nov 29;7(11):e49525. doi: 10.1371/journal.pone.0049525 (PMC3510216; doi:10.1371/journal.pone.0049525)
Supplement: Table S1 — The list of genomic regions likely to be under selection ( P <0.01, genome-wide). (DOC) [file pone.0049525.s033.doc]

**Supplementary table 1.** A descriptive summary of genomic regions identified as candidate selective sweeps in the egg laying chicken. Consecutive windows with significant reduction in pooled heterozygosity (*P*≤0.01) were merged.

|  | Chr | *H_P_^a^* | Wstart^b^ | Wend^c^ | Wsize^d^ | nSNP^e^ | *P-value* | Gene^f^ |
| --- | --- | --- | --- | --- | --- | --- | --- | --- |
| 1 | 1 | 0.219 | 14’358’278 | 14’438’278 | 37’933 | 10 | 0.001 | ORC5L |
| 2 | 1 | 0.226 | 20’734’811 | 20’814’811 | 39’865 | 13 | 0.001 |  |
| 3 | 1 | 0.237 | 31'931’307 | 32'011’307 | 36’819 | 19 | 0.001 |  |
| 4 | 1 | 0.214 | 33’022’141 | 33’102’141 | 34’903 | 14 | 0.001 | AMIGO2 |
| 5 | 1 | 0.213 | 41'874’275 | 41'954’275 | 37’282 | 10 | 0.001 | MYF6, MYF5 |
| 6 | 1 | 0.247 | 57’254’076 | 57’334’076 | 39’619 | 72 | 0.001 | IGF1 |
| 7 | 1 | 0.222 | 61’726’103 | 61’806’103 | 40’000 | 26 | 0.001 | C1QDC1 |
| 8 | 1 | 0.217 | 63’629’771 | 63’709’771 | 38’346 | 11 | 0.001 | CACNA1C |
| 9 | 1 | 0.221 | 70’862’286 | 70’942’286 | 36’970 | 17 | 0.001 |  |
| 10 | 1 | 0.243 | 92’573’625 | 92’653’625 | 39’545 | 43 | 0.001 | EPHA3 |
| 11 | 1 | 0.224 | 106’471’965 | 106’551’965 | 39’414 | 11 | 0.001 |  |
| 12 | 1 | 0.249 | 114’854’820 | 114’934’820 | 39’963 | 53 | 0.001 | MAOB, MAOA |
| 13 | 1 | 0.196 | 130’519’352 | 130’599’352 | 39’674 | 16 | 0.001 | HDHD1A |
| 14 | 1 | 0.235 | 145’563’825 | 145’643’825 | 38’269 | 29 | 0.001 |  |
| 15 | 2 | 0.245 | 9’218’712 | 9’298’712 | 38’411 | 30 | 0.001 |  |
| 16 | 2 | 0.238 | 18’225’848 | 18’305’848 | 39’937 | 32 | 0.001 | NEBL |
| 17 | 2 | 0.239 | 22’684’792 | 22’764’792 | 36’420 | 16 | 0.001 | CDK6 |
| 18 | 2 | 0.239 | 46’030’791 | 46’110’791 | 39’938 | 22 | 0.001 | MLH1, STARD3NL |
| 19 | 2 | 0.234 | 78’151’063 | 78’231’063 | 33’908 | 30 | 0.001 | TRIO |
| 20 | 2 | 0.234 | 91’259’496 | 91’339’496 | 39’545 | 17 | 0.001 |  |
| 21 | 2 | 0.249 | 117’326’532 | 117’406’532 | 39’556 | 37 | 0.001 |  |
| 22 | 2 | 0.204 | 128’895’527 | 128’975’527 | 39’569 | 65 | 0.001 |  |
| 23 | 2 | 0.237 | 133’959’036 | 134’039’036 | 38’257 | 10 | 0.001 |  |
| 24 | 2 | 0.228 | 138’174’986 | 138’254’986 | 37’891 | 24 | 0.001 |  |
| 25 | 2 | 0.238 | 140’457’287 | 140’537’287 | 30’838 | 32 | 0.001 |  |
| 26 | 3 | 0.231 | 9’914’466 | 9’994’466 | 37’578 | 38 | 0.001 | ACTR2 |
| 27 | 3 | 0.249 | 16’554’590 | 16’634’590 | 39’676 | 46 | 0.001 |  |
| 28 | 3 | 0.219 | 24’624’483 | 24’704’483 | 39’486 | 14 | 0.001 |  |
| 29 | 3 | 0.218 | 30’254’700 | 30’334’700 | 39’859 | 31 | 0.001 |  |
| 30 | 3 | 0.241 | 37’322’968 | 37’402’968 | 39’000 | 53 | 0.001 | RGS7 |
| 31 | 3 | 0.226 | 39’816’179 | 39’896’179 | 39’347 | 16 | 0.001 | TOMM20 |
| 32 | 3 | 0.229 | 96’796’884 | 96’876’884 | 37’661 | 18 | 0.001 |  |
| 33 | 3 | 0.241 | 109’565’405 | 109’645’405 | 39’778 | 56 | 0.001 | SOX7 |
| 34 | 4 | 0.240 | 10’141’892 | 10’221’892 | 39’680 | 21 | 0.001 |  |
| 35 | 4 | 0.249 | 12’534’427 | 12’614’427 | 39’855 | 70 | 0.001 | ABCB7 |
| 36 | 4 | 0.231 | 17’382’561 | 17’462’561 | 36’106 | 29 | 0.001 | MAP4K4 |
| 37 | 4 | 0.218 | 25’022’539 | 25’102’539 | 38’776 | 19 | 0.001 | CPE |
| 38 | 4 | 0.221 | 31’035’039 | 31’115’039 | 39’554 | 20 | 0.001 | ZNF330 |
| 39 | 4 | 0.241 | 39’002’148 | 39’082’148 | 34’075 | 14 | 0.001 |  |
| 40 | 4 | 0.236 | 50’552’499 | 50’632’499 | 39’935 | 25 | 0.001 | SRD5A2L2 |
| 41 | 4 | 0.226 | 59’878’154 | 59’958’154 | 38’926 | 18 | 0.001 | PDLIM5, BMPR1B |
| 42 | 5 | 0.225 | 8’648’945 | 8’728’945 | 39’690 | 14 | 0.001 | TEAD1 |
| 43 | 5 | 0.247 | 20’269’966 | 20’349’966 | 39’814 | 19 | 0.001 | APIP |
| 44 | 5 | 0.215 | 55’705’062 | 55’785’062 | 35’261 | 27 | 0.001 |  |
| 45 | 6 | 0.243 | 372’547 | 452’547 | 39’906 | 77 | 0.001 |  |
| 46 | 6 | 0.225 | 5’018’692 | 5’098’692 | 39’417 | 23 | 0.001 |  |
| 47 | 6 | 0.225 | 8’482’231 | 8’562’231 | 39’749 | 44 | 0.001 |  |
| 48 | 6 | 0.236 | 28’348’700 | 28’428’700 | 39’928 | 22 | 0.001 |  |
| 49 | 6 | 0.243 | 31’219’389 | 31’299’389 | 38’965 | 33 | 0.001 | PRLHR |
| 50 | 7 | 0.249 | 14’318’645 | 14’398’645 | 39’688 | 33 | 0.001 |  |
| 51 | 7 | 0.212 | 24’283’380 | 24’363’380 | 37’053 | 12 | 0.001 |  |
| 52 | 7 | 0.251 | 29’173’006 | 29’253’006 | 39’084 | 20 | 0.001 |  |
| 53 | 8 | 0.250 | 7’782’262 | 7’862’262 | 39’002 | 61 | 0.001 | LAMC1, LAMC2 |
| 54 | 8 | 0.221 | 15’499’535 | 15’579’535 | 38’496 | 27 | 0.001 |  |
| 55 | 8 | 0.238 | 20’464’678 | 20’544’678 | 39’179 | 31 | 0.001 |  |
| 56 | 8 | 0.210 | 26’604’752 | 26’684’752 | 38’853 | 11 | 0.001 | C8B, DAB1 |
| 57 | 9 | 0.249 | 2’469’493 | 2’549’493 | 39’402 | 28 | 0.001 | UGCGL1, HS6ST1 |
| 58 | 9 | 0.228 | 13’160’113 | 13’240’113 | 39’417 | 10 | 0.001 |  |
| 59 | 9 | 0.226 | 17’176’490 | 17’256’490 | 39’345 | 46 | 0.001 | FETUB |
| 60 | 10 | 0.242 | 3’850’374 | 3’930’374 | 39’881 | 39 | 0.001 | HMG20A |
| 61 | 10 | 0.236 | 18’569’896 | 18’649’896 | 38’306 | 49 | 0.001 |  |
| 62 | 11 | 0.249 | 10’480’229 | 10’560’229 | 35’790 | 31 | 0.001 | PDCD5 |
| 63 | 11 | 0.235 | 19’076’022 | 19’156’022 | 39’570 | 18 | 0.001 |  |
| 64 | 12 | 0.213 | 2’648’962 | 2’728’962 | 39’736 | 14 | 0.001 | USP4, RHOA |
| 65 | 12 | 0.242 | 4’573’225 | 4’653’225 | 39’374 | 56 | 0.001 | ATG7 |
| 66 | 12 | 0.240 | 6’330’339 | 6’410’339 | 39’453 | 59 | 0.001 | BARX1 |
| 67 | 13 | 0.230 | 7’211’885 | 7’291’885 | 39’546 | 25 | 0.001 | GABARB |
| 68 | 14 | 0.221 | 2’535’058 | 2’615’058 | 39’972 | 40 | 0.001 |  |
| 69 | 14 | 0.226 | 8’201’199 | 8’281’199 | 38’949 | 45 | 0.001 | XYLT1 |
| 70 | 14 | 0.250 | 13’185’993 | 13’265’993 | 39’997 | 52 | 0.001 | TRAP1 |
| 71 | 15 | 0.224 | 6’285’965 | 6’365’965 | 35’996 | 28 | 0.001 | MAPKAPK5, ADAM-35, ERP29, TRAFD1 |
| 72 | 15 | 0.262 | 11’580’805 | 11’660’805 | 39’520 | 60 | 0.001 |  |
| 73 | 17 | 0.239 | 7’442’140 | 7’522’140 | 37’888 | 27 | 0.001 | GTF3C5, GBGT1, SURF6 |
| 74 | 20 | 0.242 | 1’809’865 | 1’889’865 | 38’155 | 21 | 0.001 |  |
| 75 | 20 | 0.240 | 8’628’384 | 8’708’384 | 39’916 | 32 | 0.001 | YTHDF1 |
| 76 | 22 | 0.241 | 1’929’789 | 2’009’789 | 38’835 | 66 | 0.001 |  |
| 77 | 26 | 0.245 | 3’788’741 | 3’868’741 | 39’967 | 13 | 0.001 | TSHB, TSPAN2, NGFB |
| 78 | 26 | 0.240 | 4’874’235 | 4’954’235 | 36’601 | 17 | 0.001 | TBN, CHIA, BTG2, FMOD |
| 79 | 27 | 0.226 | 3’538’962 | 3’618’962 | 39’198 | 25 | 0.001 | MIRN196-1, HOXB8, HOXB5, HOXB3 |
| 80 | 1 | 0.268 | 7’348’168 | 7’428’168 | 39’489 | 92 | 0.01 |  |
| 81 | 1 | 0.258 | 17’874’977 | 17’954’977 | 39’460 | 13 | 0.01 |  |
| 82 | 1 | 0.258 | 49’958’517 | 50’038’517 | 39’984 | 53 | 0.01 | H3F3A, H2A-IX |
| 83 | 1 | 0.255 | 52’082’080 | 52’162’080 | 33’108 | 13 | 0.01 |  |
| 84 | 1 | 0.270 | 79’684’946 | 79’764’946 | 39’986 | 105 | 0.01 | CD86 |
| 85 | 1 | 0.252 | 84’055’225 | 84’135’225 | 33’227 | 39 | 0.01 |  |
| 86 | 1 | 0.260 | 156’983’940 | 157’063’940 | 39’910 | 44 | 0.01 |  |
| 87 | 1 | 0.269 | 163’610’378 | 163’690’378 | 39’177 | 25 | 0.01 |  |
| 88 | 1 | 0.259 | 169’626’400 | 169’706’400 | 39’747 | 63 | 0.01 |  |
| 89 | 1 | 0.251 | 173’273’802 | 173’353’802 | 39’907 | 63 | 0.01 | FNDC3A |
| 90 | 2 | 0.252 | 5’021’273 | 5’101’273 | 38’584 | 64 | 0.01 |  |
| 91 | 2 | 0.267 | 38’780’745 | 38’860’745 | 39’628 | 59 | 0.01 |  |
| 92 | 2 | 0.253 | 56’684’649 | 56’764’649 | 39’529 | 24 | 0.01 |  |
| 93 | 2 | 0.253 | 81’575’365 | 81’655’365 | 39’831 | 32 | 0.01 |  |
| 94 | 3 | 0.252 | 33’746’005 | 33’826’005 | 39’722 | 71 | 0.01 |  |
| 95 | 3 | 0.256 | 44’892’802 | 44’972’802 | 39’887 | 70 | 0.01 |  |
| 96 | 3 | 0.266 | 52’095’717 | 52’175’717 | 39’988 | 24 | 0.01 | CNKSR3 |
| 97 | 3 | 0.269 | 71’279’465 | 71’359’465 | 36’607 | 66 | 0.01 |  |
| 98 | 3 | 0.254 | 76’818’586 | 76’898’586 | 39’591 | 39 | 0.01 |  |
| 99 | 3 | 0.262 | 104’235’864 | 104’315’864 | 39’956 | 38 | 0.01 | MATN3 |
| 100 | 3 | 0.271 | 107’024’043 | 107’104’043 | 39’372 | 88 | 0.01 | FKBP1B |
| 101 | 4 | 0.254 | 2’167’410 | 2’247’410 | 38’579 | 56 | 0.01 | BRCC3, OGT, TAF1, RHOG, ITGB1BP2 |
| 102 | 4 | 0.262 | 34’722’231 | 34’802’231 | 39’591 | 86 | 0.01 |  |
| 103 | 4 | 0.253 | 47’828’631 | 47’908’631 | 39’362 | 48 | 0.01 | HNRPDL, HNRPD, HPSE |
| 104 | 4 | 0.268 | 69’153’398 | 69’233’398 | 39’361 | 84 | 0.01 |  |
| 105 | 4 | 0.270 | 76’283’903 | 76’363’903 | 39’748 | 83 | 0.01 |  |
| 106 | 5 | 0.262 | 1’457’383 | 1’537’383 | 39’763 | 46 | 0.01 | IGHMBP2, SYT12 |
| 107 | 5 | 0.265 | 6’139’562 | 6’219’562 | 39’768 | 76 | 0.01 |  |
| 108 | 5 | 0.267 | 30’076’338 | 30’156’338 | 39’406 | 73 | 0.01 |  |
| 109 | 5 | 0.254 | 36’368’700 | 36’448’700 | 39’854 | 57 | 0.01 |  |
| 110 | 5 | 0.262 | 39’607’372 | 39’687’372 | 39’948 | 54 | 0.01 |  |
| 111 | 5 | 0.252 | 43’346’097 | 43’426’097 | 39’921 | 70 | 0.01 | SEL1L |
| 112 | 6 | 0.256 | 11’846’215 | 11’926’215 | 37’768 | 72 | 0.01 | CCAR1 |
| 113 | 7 | 0.250 | 1’720’364 | 1’800’364 | 39’600 | 48 | 0.01 | ZNF804A |
| 114 | 7 | 0.252 | 6’268’010 | 6’348’010 | 39’235 | 45 | 0.01 | HDAC4 |
| 115 | 7 | 0.254 | 26’874’066 | 26’954’066 | 39’983 | 55 | 0.01 |  |
| 116 | 8 | 0.251 | 4’148’098 | 4’228’098 | 39’526 | 59 | 0.01 | PACS2, SEC22B, NOTCH2 |
| 117 | 9 | 0.255 | 387’275 | 467’275 | 39’765 | 33 | 0.01 |  |
| 118 | 9 | 0.266 | 5’786’682 | 5’866’682 | 38’487 | 44 | 0.01 | GAL3ST2, D2HGDH, ING5, ATG4B |
| 119 | 9 | 0.245 | 20’500’029 | 20’580’029 | 39’804 | 30 | 0.01 |  |
| 120 | 10 | 0.259 | 1’719’115 | 1’799’115 | 39’474 | 80 | 0.01 | MUC1,CSK |
| 121 | 10 | 0.271 | 10’497’681 | 10’577’681 | 37’069 | 51 | 0.01 | CYP19A1 |
| 122 | 10 | 0.252 | 16’200’845 | 16’280’845 | 39’834 | 27 | 0.01 | ST8SIA2 |
| 123 | 11 | 0.269 | 13’729’458 | 13’809’458 | 39’954 | 57 | 0.01 |  |
| 124 | 12 | 0.259 | 13’114’003 | 13’194’003 | 39’858 | 41 | 0.01 | PTPRG |
| 125 | 12 | 0.262 | 17’074’495 | 17’154’495 | 37’788 | 19 | 0.01 | SHQ1, PPP4R2 |
| 126 | 17 | 0.251 | 3’162’947 | 3’242’947 | 39’957 | 35 | 0.01 |  |
| 127 | 18 | 0.261 | 8’333’036 | 8’413’036 | 38’992 | 33 | 0.01 |  |
| 128 | 20 | 0.257 | 6’874’355 | 6’954’355 | 39’811 | 62 | 0.01 |  |
| 129 | 23 | 0.227 | 2’200’819 | 2’280’819 | 39’984 | 31 | 0.01 |  |
| 130 | 24 | 0.260 | 2’975’604 | 3’055’604 | 39’813 | 112 | 0.01 |  |
| 131 | 28 | 0.271 | 3’411’157 | 3’491’157 | 39’849 | 51 | 0.01 |  |
| 132 | 41 | 0.263 | 579’730 | 659’730 | 38’292 | 20 | 0.01 |  |

^a^The lowest pooled heterozygosity observed for a <40 kb window in the region. ^b^starting, ^c^ending and ^d^length of the corresponding window in base pair, ^e^number of SNPs in window and ^f^gene(s) overlapping the putative sweep region with 20 kb extension from both sides.
